# Predicting atezolizumab response in metastatic urothelial carcinoma patients using machine learning on integrated tumour gene expression and clinical data

**DOI:** 10.1038/s41698-025-00969-8

**Published:** 2025-06-10

**Authors:** Chayanit Piyawajanusorn, Ghita Ghislat, Pedro J. Ballester

**Affiliations:** 1 Department of Bioengineering, Imperial College London, London, UK; 2 Princess Srisavangavadhana Faculty of Medicine, Bangkok, Thailand; 3 Department of Life Sciences, Imperial College London, London, UK

**Keywords:** Predictive markers, Computational biology and bioinformatics

## Abstract

Atezolizumab is a treatment for metastatic urothelial carcinoma (mUC), yet only 23% of mUC patients benefit from it. Worse yet, accurately predicting such responders remains challenging, despite existing biomarkers. Here we employed eight machine learning (ML) algorithms to predict mUC patient response to atezolizumab using tumours’ gene expression profiling and clinical data from two independent cohorts. The CART-OMC model developed on the discovery dataset achieved the highest performance, with a validation set Matthews correlation coefficient (MCC) of 0.437, using the expressions of just 29 ML-selected genes, including CXCL9 and IFNG. Univariate biomarkers like TMB, TNB, and PD-L1 were less predictive with MCCs of 0, 0.316, and 0, respectively. Upon merging these datasets, the best-performing model (LGBM-OMC; MCC of 0.252) also outperformed top modelling approaches such as EaSIeR (MCC ~ 0) and JADBio (MCC of 0.179). We make these promising ML models freely available to predict atezolizumab response in other mUC patients.

## Introduction

Urothelial carcinoma (UC) is the sixth most common type of cancer occurring in the urinary tract system. Its incidence is steadily increasing with an estimated 500,000 new cases and 200,000 deaths worldwide (31.7%) in 2020^1^. In total, 15% of patients are diagnosed with metastatic urothelial carcinoma (mUC), which has a 5-year survival rate of just 5%^2,3^. For decades, platinum-based chemotherapy has been the standard first-line therapy for mUC patients^4^. Despite this drug treatment improving clinical outcomes, its toxicity has been a major clinical concern. Over 60% of mUC patients are not eligible for this treatment due to poor patient functional ability assessed by Eastern Cooperative Oncology Group (ECOG) performance status or other comorbidities (e.g., advanced age, renal impairment, hearing loss and heart failure)^5,6^.

To address this clinical need, immunotherapy has emerged as a promising alternative for mUC patients who have progressed or are ineligible for platinum-based chemotherapy. The U.S. Food and Drug Administration (FDA) has successfully approved three different categories of immune checkpoint inhibitors (ICIs): PD-1 inhibitors (Nivolumab, Pembrolizumab, and Cemiplimab), PDL-1 inhibitors (Atezolizumab, Durvalumab and Avelumab) and CTLA-4 inhibitors (Ipilimumab)^7^. Atezolizumab is a monoclonal immunoglobulin G1 (IgG) antibody that binds to PD-L1 on the surface of tumour cells and other PD-L1-expressing cells, such as some tumour-infiltrating immune cells (TICs), e.g. dendritic cells. This binding blocks PD-L1 interaction with PD-1 receptors, which are mainly expressed on T cells, thereby restoring T cell-mediated anti-cancer activity^8^. Although atezolizumab has considerably improved patient outcomes in various cancer types, including mUC^8–12^ most patients do not respond, experience immune toxicities at considerable cost, and lack robust biomarkers. In the phase II IMvigor210 trial^2^, the safety and efficacy of atezolizumab in mUC were first evaluated in patients who had progressed during or following platinum-based chemotherapy. The objective response rate (ORR) of these patients, defined as the proportion of patients who achieved either a complete or partial response to treatment, was only 23% (95% CI: 16–31), with merely 9% of them exhibiting a complete response. In addition, treatment-related adverse events occurred, leading to treatment discontinuation. Therefore, there is an urgent need for predictive markers able to anticipate which mUC patients benefit the most from ICI therapy. Doing so will enable the administration of promising drugs to eligible patients without delay while minimising the risk of adverse effects for the patients unlikely to respond to ICIs^13^.

Most studies on ICI response have been limited to univariate markers. Positive PD-L1 expression, high tumour mutational burden (TMB), and high tumour neoantigen burden (TNB) have been proposed as biomarkers that could potentially predict ICI response in multiple cancer types^14–19^ However, the cutoffs for PD-L1 expression vary within and across cancer types and lack a standard PD-L1 detection assay^20^. While the measurement of TMB and TNB is challenging, costly, and time-consuming, there is also a lack of consensus on the methodologies used to consistently quantify TMB and TNB across different sequencing platforms and laboratories. Furthermore, the lack of robust cutoffs for identifying a high burden varies across cancer types^21,22^. These present significant limitations to the use of PD-L1, TMB and TNB as univariate markers in clinical practice^23^.

Recent technological advancements in high-throughput sequencing and drug sensitivity screenings have generated an abundance of data yet to be exploited^24^, driving the exploration of statistical methods^25^ or machine learning (ML) for drug response prediction in cell lines^26–28^ patient-derived xenografts (PDXs)^29^, and even cancer patients^30–32^ ML-based model can identify predictive combinations of features in high dimensional data such as genomic, transcriptomic, and imaging, unlike traditional statistical approaches (e.g. Cox regression), which assess a small number of predictors^33–35^ Few studies have demonstrated the benefits of using ML-based models to predict ICI response in cancer patients^36–39^ Nassar et al.^38^ reported a multivariable logistic regression model that combined clinical and genomic features to predict the response to anti-PD1/anti-PD-L1 therapy among 62 mUC patients. However, the results require further validation due to the limited sample size and the lack of an independent validation cohort. Despite ICIs demonstrating meaningful antitumor activity, patient response to ICIs may be influenced by various biological factors, including complex interactions in the tumour microenvironment (TME)^40^. Lapuente-Santana et al. developed an estimate systems immune response (EaSIeR), an ML approach employing multi-task ML based on system-based signatures of the TME, to predict anti-PD1/anti-PD-L1 response^39^. While EaSIeR is a valuable advance, it reported an area under the receiver operating characteristic curve (ROC-AUC) of just 0.65 when tested on the mUC cohort^39^, which shows the need for more predictive models.

To our knowledge, ML has yet to be applied to predict mUC patient response to atezolizumab. With this purpose, we retrospectively collected gene expression profiles and clinical data of mUC patients treated with atezolizumab from the IMVigor210 phase II clinical trial and Snyder studies^2,41^. We aim to develop a robust ML model that could classify atezolizumab responders and non-responders in mUC patients based on their gene expression profiles and clinical data. Since the predictive ability of ML algorithms is problem-dependent and cannot be anticipated^42^, it is important to evaluate a range of algorithms in predicting atezolizumab-treated mUC patient response.

## Results

**Fig. 1:**
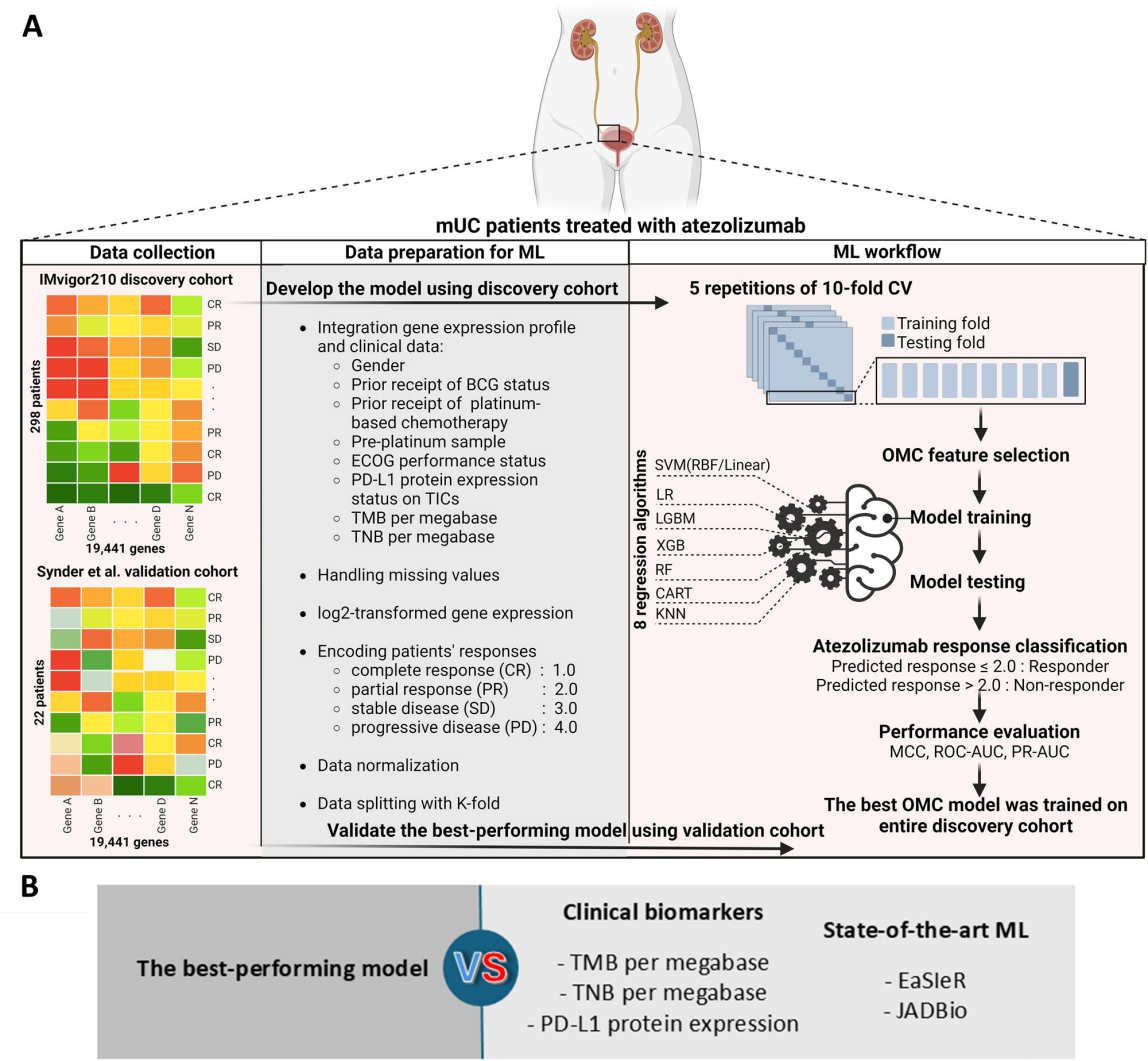
Machine learning workflow for predicting response to atezolizumab in mUC patients. **A** Schematic illustrations of developing and validating supervised ML models using gene expression profiles of mUC patient tumours treated with atezolizumab, along with clinical data (Table 1). The IMVigor210 phase II clinical trial cohort (298 patients) served as the discovery dataset for model development, while the Synder et al. cohort (22 patients) was used as a validation dataset for model validation. Both datasets were subsequently pre-processed for ML. Each of the eight regression algorithms was used to develop an ML model with optimal model complexity (OMC) feature selection, which was applied to reduce the model features from the initially considered 20,000 features. The model performance was evaluated by 10-fold cross-validation (CV) with five repetitions, each with a different random seed. The predicted responses were classified into responders or non-responders using a threshold of 2.0. In this way, the model accessed all the information about patients’ response labels (C.R., P.R., S.D. and P.D.), thus capturing the full granularity of data, rather than grouping them (e.g., C.R. and P.R. as responders, and S.D. and P.D. as non-responders) prior to modelling. The best OMC model, the one with the highest MCC, was selected and applied to the validation dataset. MCC, ROC-AUC and PR-AUC were reported. **B** The performance of the best-performing model was compared against clinical biomarkers (e.g. TMB per megabase, TNB per megabase and PD-L1 expression), as well as state-of-the-art ML models (e.g. EaSIeR and JADBio) for atezolizumab response prediction.

By using gene expression profiles and clinical data from 298 atezolizumab-treated mUC patients in the IMvigor210 discovery dataset^2^, we developed OMC models with eight regression algorithms to predict atezolizumab response. The best OMC models were then validated on 22 mUC patients treated with atezolizumab from the Snyder et al. validation dataset^41^. The baseline clinical characteristics of mUC patients with known atezolizumab response from the discovery and validation dataset are shown in Table 1. Figure 1 summarises the computational framework, which is fully described in the ‘Methods’ section.

**Table 1:** The baseline clinical characteristics of mUC patients from the discovery and validation cohorts

Characteristics	ML input	Discovery dataset (*N* = 298)	Validation dataset (*N* = 22)
Dataset		IMVigor210 phase II clinical trial	Snyder et al., 2017
Response			
Complete response (CR)	1	25 (8%)	2 (9%)
Partial response (PR)	2	43 (14%)	5 (23%)
Stable disease (SD)	3	63 (21%)	5 (23%)
Progression disease (PD)	4	167 (56%)	10 (45%)
Response rate	1	23%	32%
Gender			
Male	0	233 (78%)	20 (91%)
Female	1	65 (22%)	2 (9%)
ECOG performance status			
0	0	121 (41%)	1 (5%)
1	1	165 (55%)	21 (95%)
2	2	12 (4%)	0 (0%)
PD-L1 protein expression status on TICs			
IC0	0	83 (28%)	3 (14%)
IC1	1	112 (38%)	11 (50%)
IC2	2	102 (34%)	8 (36%)
NA	–	1 (<1%)	0 (0%)
TMB per megabase			
Number of patients	Real values	234	21
Range (Median ± SD)		0–62 (8 ± 9.38)	0–11 (1 ± 3.12)
TNB per megabase			
Number of patients	Real values	216	21
Range (Median ± SD)		0.04–11.69 (0.86 ± 1.60)	0–39(3 ± 9.17)
Prior receipt of intravesical administration of BCG immunotherapy status			
Yes	1	231 (78%)	8 (36%)
No	0	67 (22%)	14 (64%)
Received platinum-based chemotherapy			
Yes	1	233 (78%)	19 (86%)
No	0	65 (22%)	3 (14%)
Sample collected pre-platinum			
Yes	1	197 (66%)	10 (45%)
No	2	95 (32%)	9 (41%)
NA	–	6 (2%)	3 (14%)

*ECOG* Eastern Cooperative Oncology Group, *TICs* tumour-infiltrating immune cells, *TMB* tumour mutation burden, *TNB* tumour neoantigen burden, *BCG* intravesical Bacillus Calmette–Guérin.

The following were used as clinical features in ML models: gender, ECOG performance status, PD-L1 expression on TIC status, TMB per megabase, TNB per megabase, received BCG, received platinum-based chemotherapy and sample collected pre-platinum.

### Predicting mUC patient response to atezolizumab from gene expression data

**Fig. 2:**
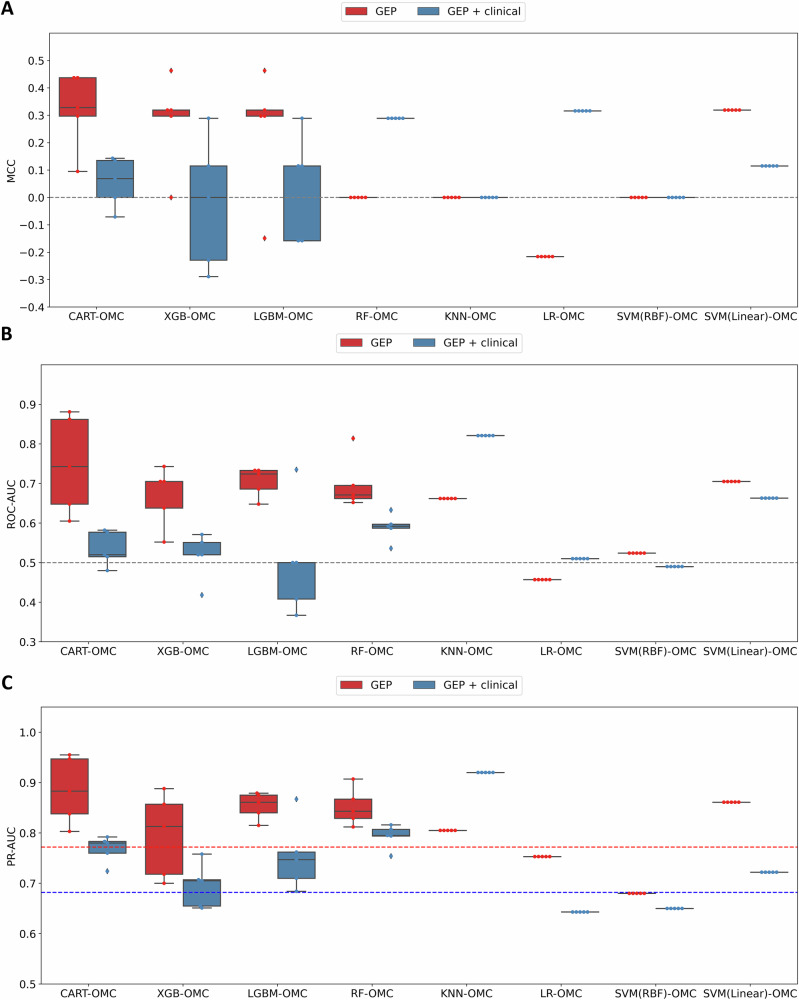
(Related to Table S3). Five-repetition evaluation metrics of the validation dataset across eight ML models using either gene expression profiles or integrated gene expression profiles plus clinical data. (**A**) MCC, (**B**) ROC-AUC and (**C**) PR-AUC are calculated using predictions on the validation dataset of the best optimal model complexity (OMC) model. The best OMC model, selected by the highest MCC from a standard 10-fold CV on the discovery dataset, was retrained on the entire discovery dataset and tested on the validation dataset (the CV is performed five times, changing the random seed to assess robustness). Eight regression algorithms using OMC feature selection to select only a small subset of informative features facilitate model prediction. Two sets of features were employed per learning algorithm: gene expression profiles (GEP) and integrated gene expression profiles with clinical data (GEP + clinical). Random-level performance is delimited by the horizontal dashed lines (0.0 for MCC, 0.5 for ROC-AUC and 0.772 and 0.682 for PR-AUC when using GEP or GEP + clinical features, respectively).

The evaluation metrics on the validation dataset across eight ML models using either gene expression profiles (GEP) is shown in Fig. 2. A 10-fold CV was run on the discovery dataset to select the best OMC model, which was subsequently tested on the validation dataset (the CV is performed 5 times changing the random seed to assess robustness). In the validation dataset, four models using GEP distinguish responders from non-responders with median MCC (mMCC) over 0.2: CART-OMC (mMCC of 0.328), XGB-OMC (mMCC of 0.319), SVM (Linear)-OMC (mMCC of 0.319) and LGBM-OMC (mMCC of 0.297). CART-OMC achieved the highest mMCC by non-linearly combining 29 genes out of 19,441 genes (0.15%) as predictive features for atezolizumab-treated mUC patient response prediction. The list of these 29 predictive genes is shown in Table 2.

**Table 2:** The list of 29 predictive genes derived from the best-performing model (CART-OMC) employed gene expression profiles

Entrez gene	Gene symbol	Gene name
2822	GPLD1	Glycosylphosphatidylinositol specific phospholipase D1
2835	GPR12	G protein-coupled receptor 12
3458	IFNG	Interferon gamma
3627	CXCL10	C–X–C motif chemokine ligand 10
3682	ITGAE	Integrin subunit alpha E
3766	KCNJ10	Potassium inwardly rectifying channel subfamily J member 10
3805	KIR2DL4	Killer cell immunoglobulin-like receptor, two Ig domains and a long cytoplasmic tail 4
3822	KLRC2	Killer cell lectin-like receptor C2
3823	KLRC3	Killer cell lectin-like receptor C3
3902	LAG3	Lymphocyte activating 3
4283	CXCL9	C-X-C motif chemokine ligand 9
4319	MMP10	Matrix metallopeptidase 10
5540	NPY4R	Neuropeptide Y receptor Y4
6273	S100A2	S100 calcium-binding protein A2
8302	KLRC4	Killer cell lectin-like receptor C4
9437	NCR1	Natural cytotoxicity triggering receptor 1
10563	CXCL13	C–X–C motif chemokine ligand 13
10578	GNLY	Granulysin
54436	SH3TC1	SH3 domain and tetratricopeptide repeats 1
56896	DPYSL5	Dihydropyrimidinase like 5
57149	LYRM1	LYR motif containing 1
59067	IL21	Interleukin 21
81691	REXO5	RNA exonuclease 5
83546	RTBDN	Retbindin
84630	TTBK1	Tau tubulin kinase 1
121643	FOXN4	Forkhead box N4
140767	NRSN1	Neurensin 1
257101	ZNF683	Zinc finger protein 683
340562	SATL1	Spermidine/spermine N1-acetyl transferase like 1

### Predicting mUC patient response to atezolizumab from gene expression and clinical data

We investigated whether combining gene expression profiles with clinical data improves atezolizumab response prediction in mUC patients. The clinical features included gender, ECOG performance status, PD-L1 protein expression status on TICs, TMB per megabase, TNB per megabase, prior receipt of intravesical administration of Bacillus Calmette–Guérin (BCG) immunotherapy status, platinum-based chemotherapy status, and pre-platinum sample collection status (Table 1). Because TMB, TNB and PDL1 are the up-to-date most relevant biomarkers of ICI therapy, we detail here how the technical and methodological differences between the discovery and validation datasets for these three features. Both the discovery and validation datasets reported TMB and TNB as mutations per megabase, normalised to account for differences in gene panel size and the target region. However, detection methodologies, calculation methods and other bioinformatics test specifications vary across laboratories, contributing to data inconsistencies. In calculating TMB and TNB, the discovery dataset^2^ considered all somatic coding mutations, including synonymous and non-synonymous, per megabase of the tumour genome examined by the FoundationOne CDx DNA-based commercial panel. By contrast, the validation dataset^41^ considered only non-synonymous mutations from the IDT xGen Whole Exome Panel. Although both studies estimated neoantigens using non-synonymous mutations, they utilised different bioinformatics tools for neoantigen prediction. On the other hand, PD-L1 expression on TICs was identically assessed in both studies, using the SP142 immunohistochemistry assay, categorising tumours as IC0 (<1%), IC1 (≥1% and <5) and IC2/3 (≥5%) based on the percentage of PD-L1-positive TICs.

The evaluation metrics on the validation dataset for the models trained in the discovery dataset using GEP plus clinical features are presented in Fig. 2. Only two models were able to distinguish between responders and non-responders with an mMCC of at least 0.2: LR-OMC (mMCC of 0.316) and RF-OMC (mMCC of 0.289). LR-OMC achieved the highest mMCC, which linearly combined 67 genes with TMB and TNB among 19,449 features (0.35%) for atezolizumab response prediction, referred to as GEP + TMB + TNB (LR-OMC). The list of these 69 predictive features is shown in Table S4.

**Fig. 3:**
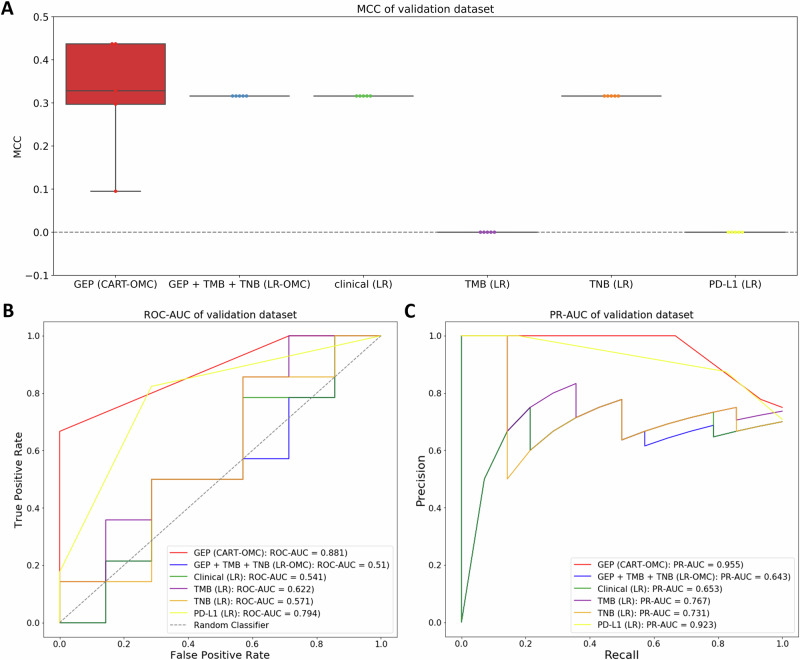
Five-repetition evaluation on the validation dataset of the best-performing models versus those using TMB, TNB or PD-L1 immunotherapy biomarkers. **A** MCCs of five models (a different random seed per repetition), **B** ROC-AUC corresponding to the run with median MCC and **C** PR-AUC corresponding to the run with median MCC. Each metric is calculated using predictions on the validation dataset of the ML model built on the discovery dataset using gene expression profiles (GEP), integrated gene expression profiles with clinical data (GEP + clinical), referred to GEP + TMB + TNB, clinical data only (Table 1) (clinical), TMB per megabase (TMB), TNB per megabase (TNB) and PD-L1 protein expression on tumour-infiltrating immune cells (PD-L1). The CV is performed five times, changing the random seed, and each dot represents a repetition shown with a boxplot. Random-level performance is delimited by the horizontal dashed lines (0.0 for MCC, 0.5 for ROC-AUC, 0.772 for PR-AUC when using GEP and 0.682 for PR-AUC when using GEP + TMB + TNB, clinical data only, TMB, TNB or PD-L1).

However, the prediction based on combined clinical-gene expression profiles, GEP + TMB + TNB (LR-OMC) with mMCC of 0.316, and the baseline clinical features only, clinical (LR-OMC) with mMCC of 0.316, was worse than using only gene expression profiles alone, GEP(CART-OMC) with mMCC of 0.328 (Fig. 3). Therefore, there is no benefit in integrating patients’ clinical data with gene expression profiles in predicting the atezolizumab response of mUC patients.

### Predicting mUC patient response to atezolizumab from existing immunotherapy markers

It has been reported that TMB and TNB are associated with immunotherapy response prediction in several cancer types, including mUC, with high TMB and TMB frequency associated with drug sensitivity^19,43–45^, Additionally, PD-L1 protein expressed on the surface of either tumours or TICs is an atezolizumab target given the mechanism of interaction between PD-1 and PD-L1 and emerges as an early biomarker to be tested in immunotherapy clinical trials^46,47^, To compare the predictive performance at this task, LR was trained on the normalised TMB per megabase, TNB per megabase, and PD-L1 protein expression status on TICs. Figure 3 shows that CART-OMC, nonlinearly combining 29 genes (highest MCC of 0.437), outperformed LR prediction using only TMB (MCC of 0.000), TNB (MCC of 0.316) and PD-L1 protein expression on TICs (MCC of 0.000). The results suggest that the prediction-based 29 predictive genes outperform those of a single immunotherapy biomarker in predicting atezolizumab responses.

### Analysis of cluster heat map using the 29 predictive genes

Here, there is a total of 374 mUC patients with gene expression profiles available in the discovery (*N* = 348) and validation (*N* = 26) datasets, including those with known atezolizumab response (discovery: *N* = 298, validation: *N* = 22). We aimed to assess whether the subset of patients with known responses and gene profiling from the discovery datasets used for model development could represent all the remaining patients, which could serve as the potential test set. Figure S1 shows how a total of 374 patients cluster by their similarity in the expression of 29 predictive genes identified by the best-performing CART-OMC model, using agglomerative hierarchical clustering with Ward’s linkage. A dendrogram heatmap, generated using Seaborn (version 0.11.2), shows that patients from the validation dataset and those with unknown atezolizumab response records were evenly distributed across clusters. This indicates that the model’s predictions were not limited to a specific subset of patients. Instead, the model’s applicability domain was broad and could provide accurate predictions for all these patients.

### Importance and differential expression of the 29 predictive genes

We next investigated the importance of 29 predictive genes from the best-performing model, GEP (CART-OMC), to estimate the contribution of each gene to model predictive accuracy. In the case of CART-OMC, this is computed as the reduction of Gini impurity of each feature in the decision tree^48^. The feature importance plot (Fig. S2) revealed that CXCL9 is the most important predictor of atezolizumab response, which appears in the common features between the 29 genes from the GEP validation analyses and the 49 genes from GEP merged analyses (Ven diagram in Table S5). GPR12 was the second most important gene, showing a level of importance similar to FOXN14, while some other genes had near-zero importance. These genes contributed less to the CART-OMC model prediction, but seemingly might still carry valuable information when combined with other features. For example, IFNG induces the production of CXCL9 and CXCL10^49^.

However, a feature importance plot does not indicate whether a gene positively or negatively impacts predictions. To investigate this question, we used the Shapley additive explanations (SHAP) values, which here relate gene expression levels to their impact on the model’s prediction. In Figure S3, genes were ranked by mean absolute SHAP values across all patients, with positive values increasing the likelihood of non-response and negative values indicating a higher chance of response. CXCL9 remained the most important feature, with the highest mean absolute SHAP values of 0.245, and higher expression correlated with predicted responders. Most genes showed higher expression in predicted responders, consistent with the differential expression patterns in Fig. S4. In the discovery dataset, 27 genes had significantly higher expression in responders (except for MMP10 and SH3TC1). A similar trend toward high expression in responders could be observed in the validation dataset.

### PPI network analysis of the 29 predictive genes

We next analyse the physical and functional associations between 29 predictive genes and the CD274 gene, which codes for PD-L1 and hence could influence atezolizumab response in mUC patients. We performed PPI analysis using Search Tool for Retrieval of Interacting Genes/Proteins (STRING) database version 11.5 (https://string-db.org)^50^, which integrates both known and predicted PPIs. Figure S5 illustrates the resulting network with 30 nodes (protein-coding genes) and 54 edges (interactions), with significant PPI enrichment (*p*-value < 0.0001). This indicates that the proteins in the input set have more interactions among themselves than would be expected for a randomly drawn set of proteins of the same size and degree distribution from the genome. The known and predicted interactions were observed among 15 genes, while the remaining genes were disconnected nodes in the network. Interestingly, eight genes (CXCL13, CXCL10, CXCL9, IFNG, NCR1, GNLY, LAG3 and ITGAE) were directly connected to CD274, suggesting that their roles in immune cell activation and migration, leading to tumour suppression, are influenced by CD274 signalling after PD-L1 is bound by atezolizumab. In addition, IFNG interacted with several other genes, including CD274, supporting the well-established role of IFNG as a key player in inflammatory immune responses^49^. Overall, these findings highlight molecular interactions that could contribute to atezolizumab response and suggest potential targets for improving treatment efficacy in mUC patients.

### Wikipathway cancer and GO pathway enrichment analysis of the 29 predictive genes

We next identified biological pathways that were significantly enriched with a subset of 29 predictive genes using the cancer-related repository of WikiPathways (WikiPathway cancer) and Gene Ontology (GO) database in the WEB-based Gene SeT AnaLysis Toolkit (WebGestalt) 2024 (www.webgestalt.org)^51^. One cancer-associated pathway was significantly enriched using WikiPathway cancer (Fig. S6, Table S6). Specifically, three TME-associated genes (IFNG, CXCL9 and CXCL10) were significantly enriched (FDR ≤ 0.05) in the type II interferon signalling (IFNG) pathway, which is associated with the atezolizumab mechanism. These genes encode IFNG cytokine, CXCL9 and CXCL10 chemokines produced by T cells, natural killer (NK) cells and macrophages to attach and activate immune cells for eliminating cancer cells when PD1/PD-L1 interaction is blocked by atezolizumab^52,53^, Therefore, increased intratumoral production of IFNG, CXCL3 and CXCL10 directly suppresses tumour cell proliferation and increases antigen presentation, leading to better recognition and elimination of cancer cells^54^. IFNG is considered a crucial marker in predicting immunotherapy response. Ayers et al.^55^ analysed gene expression profiles of tumour tissue samples and found that patients with metastatic melanoma, head and neck squamous cell carcinoma, and gastric cancer who responded to anti-PD1 therapy exhibited higher expression scores for IFNG-related genes compared to non-responders. The authors suggested that the identified IFNG signature (IDO1, CXCL10, CXCL9, HLA-DRA, STAT1 and IFNG) could serve as a predictive marker for the clinical response to ICIs.

GO terms, including biological process, cellular component, and molecular function pathways, were significantly enriched (FDR ≤ 0.05), with a subset of 29 genes presented in Fig. S6 and Table S6. The enriched biological process terms were involved in immunotherapy response mechanisms, such as cellular defence response, immune cell-mediated immunity and cytotoxicity, cell killing and regulation of immune system process and immune response. These pathways play a critical role in the immune system’s response to cancer, involving the production of cytokines, inflammation and recruiting T-cells and other immune cells to the TME^56^. Similar to the enriched pathways in molecular function terms, chemokine and cytokine receptor binding pathways lie in their ability to recruit and activate immune cells to attack cancer cells, promote the production of inflammatory cytokines and induce an anti-tumour immune response. The binding of CXCL9, CXCL10 and CXCL11 to CXCR3 induces T-cell and NK cell migration to the TME, where they can recognise and eliminate cancer cells^52^. Furthermore, the cellular component term showed the external side of the plasma membrane was enriched with five genes (CXCL10, CXCL9, ITGAE, LAG3 and RTBDN), indicating the site of interaction between PD-L1 expressed on the surface of the tumour or TICs and atezolizumab^57^.

### Comparison of the best-performing ML models to EaSIeR models

We have so far evaluated models on the validation set, which is robust in that this is an independent patient cohort. However, it is a small set and thus there is the risk that the models with the best validation results may not generalise well to other cohorts. Thus, we merged the discovery and validation datasets, resulting in 320 atezolizumab-treated mUC patients (75 responders, 245 non-responders), to obtain out-of-sample predictions for a much more representative set of patients. By using gene expression profiles alone or integrated with clinical data, we built OMC models to predict atezolizumab response from merged discovery and validation datasets. We evaluated eight ML algorithms through five nested 10-fold CV runs (the CV is performed 5 times, changing the random seed), with the best-performing model determined by the highest MCC. The model’s performance was compared with state-of-the-art ML drug response prediction models (e.g. EaSIeR and JADBio). Using gene expression profiles, the LGBM-OMC model, referred to as GEP (LGBM-OMC), achieved the highest MCC of 0.252, which nonlinearly combined 49 genes. Nineteen of these genes overlapped with the 29 genes from the best-performing CART-OMC model in validation analyses (Table S5). The CART-OMC also ranked among the top models in the merged datasets. The CART-OMC on integrated expressions of 63 genes, TMB and TNB features, called GEP + TMB + TNB (CART-OMC), performed slightly better than those using gene expression profiles alone, with the highest MCC of 0.253 (Fig. S7). Among 65 features identified in merged analyses, 24 overlapped with the 69 features from the best-performing LR-OMC model in the validation set (Table S5).

**Fig. 4:**
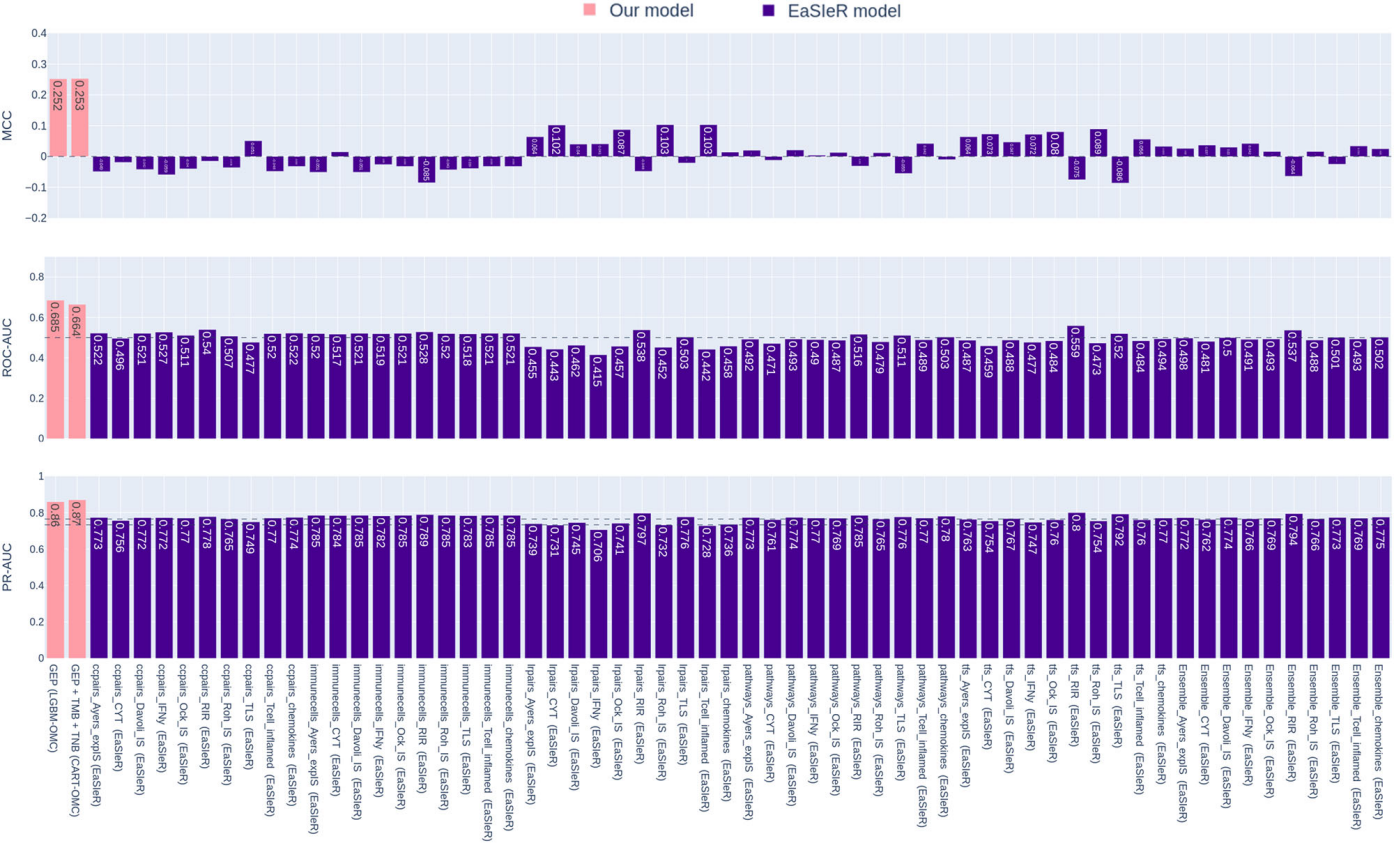
Performances of the best-performing atezolizumab-response-prediction models versus those from the 60 EaSIeR model predictions on the 320 mUC patients from the merged discovery and validation datasets (245 non-responders and 75 responders). The first two columns on the left are the best-performing models built in this study with either gene expression profiles, GEP (LGBM-OMC), or integrated gene expression profiles with clinical data, GEP + TMB + TNB (CART-OMC), using nested 10-fold CV. The CV is performed 5 times, changing the random seed, and the one with the highest MCC is shown in the bar plot. Whereas the other 60 columns on the right are the results of running the EaSIeR models on the same dataset. BLCA-specific elasticnet regularised multi-task linear regression (RMTL) models, built on TCGA-BLCA samples available in EaSIeR to predict 10 tasks of immune response scores (CYT, Ock_IS, Roh_IS, Chemokine, Davoli_IS, IFNy, Ayer_expIS, Tcell_inflamed, RIR, TLS), were used to predict the patient likelihood of response to atezolizumab based on individuals or a combination (ensemble) of five system-based signatures of TME, including cell–cell pairs (ccpairs), immune cell types (immunecells), ligand–receptor pairs (lrpairs), pathway activities (pathways), and transcriptional factor activities (tfs), producing a total of 60 EaSIeR model predictions (named by adding the suffix of immune response scores to the names of system-based signatures of TME e.g. ccpairs_CYT). Random-level performance is delimited by horizontal dashed lines (0.0 for MCC, 0.5 for ROC-AUC, 0.765 for PR-AUC when using GEP and 60 EaSIeR models, 0.733 for PR-AUC when using GEP + TMB + TNB). Overall, the best-performing models (pink) outperformed all of the 60 EaSIeR models (violet).

To predict atezolizumab response in the same set of 320 mUC patients using EaSIeR, RNA-sequencing data were input to quantify patient-specific, five real-valued system-based signatures of TME, including 11 immune cell types, 14 pathway activities, 118 transcriptional factor activities, 812 ligand-receptor pairs and 169 cell-cell pairs. These signatures allow a comprehensive characterisation of tumours, highlighting how the complex cellular composition of TMEs, inter- and intracellular mechanisms are involved in mediating the immune response and affecting immunotherapy efficacy. EaSIeR includes BLCA-specific RMTLR models exploiting TCGA-BLCA samples. Since mUC often originates from bladder cancer, these models were used to predict 10 tasks of immune response scores (CYT, Ock_IS, Roh_IS, Chemokine, Davoli_IS, IFNy, Ayer_expIS, Tcell_inflamed, RIR, TLS; Table S1) in mUC patients. 10 scores were predicted based on 6 sets of features, including five system-based signatures of TME and their combination, producing a total of 60 EaSIeR model predictions for atezolizumab response from the merged discovery and validation datasets. The evaluation metrics demonstrate the performance of our best-performing models in predicting atezolizumab response for 320 mUC patients, compared to 60 EaSIeR model predictions (Fig. 4). EaSIeR model predictions varied across different models, with MCCs near-random predictive levels. Our best-performing models built in this study, GEP(LGBM-OMC) and GEP + TMB + TNB(CART-OMC), outperformed all 60 EaSIeR model predictions, indicating they are more predictive than TME-based biomarkers from EaSIeR models in predicting atezolizumab response in mUC patients.

### Enhancing prediction on the merged dataset by integrating TME descriptors, immune response scores and the EaSIeR score

**Fig. 5:**
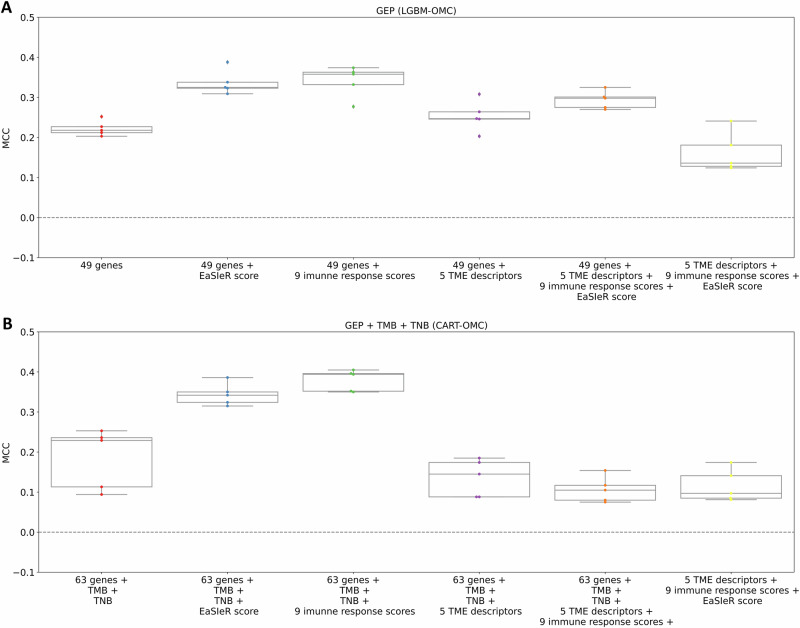
Comparison of the predictive performance of the best-performing models using their predictive features, integrated with TME descriptors, immune response scores and the EaSIeR score for atezolizumab response prediction in 320 mUC patients from the merged discovery and validation datasets. Boxplots comparing MCCs obtained from five 10-fold CV runs (each dot represents a run) of the best-performing models: LGBM combined 49 predictive genes (**A**), and CART-OMC combined 63 genes with TMB and TNB (**B**), to those combining with five TME descriptors, nine published immune response scores (Ock_IS is not available in the EaSIeR tool), and EaSIeR score. These features account for the cellular composition of TMEs together with inter- and intracellular communication, offering a comprehensive view of tumour-immune interactions affecting the efficacy of immunotherapy. Additionally, immune response scores and the EaSIeR score are proposed as predictors of response to ICIs. Random-level performance is delimited by the horizontal dashed lines at an MCC of 0.0. Overall, integrating five TME descriptors, nine immune response scores, and the EaSIeR score into the predictive model improved the predictive performance compared to those built solely on predictive genes. Specifically, nine immune response scores contributed significantly to improving the accuracy of both LGBM and CART models.

After identifying LGBM-OMC combining 49 genes (GEP (LGBM-OMC)), and CART-OMC combining 63 genes with TMB and TNB (GEP + TMB + TNB (CART-OMC)) as the best-performing models, we next looked at whether adding 1134 features computed for each patient using EaSIeR, including five system-based signatures of TMEs, referred to as TME descriptors (1,124 features), 9 published immune response scores (Ock_IS is not available in the EaSIeR tool), and the EaSIeR score, could improve prediction accuracy. The EaSIeR score is the average of 10 immune response predictions based on ensemble TME descriptors. Figure 5A shows that adding individual sets of features (TME descriptors, immune response scores, and the EaSIeR score), or their combinations, to the 49-gene LGBM model improved predictive performance compared to those built solely on 49 genes (mMCC of 0.218). The best performance came from combining 49 genes with 9 immune response scores (mMCC of 0.358), while using only a combination of five TME descriptors, 9 immune response scores and the EaSIeR score resulted in the lowest performance (mMCC of 0.136).

Similarly, Fig. 5B shows that CART with 63 genes, TMB, and TNB, when combined with either the EaSIeR score (mMCC of 0.342) or 9 immune response scores (mMCC of 0.394), improved predictive performance compared to those built solely on 63 genes, TMB and TNB (mMCC of 0.236). CART was not able to cope with the higher number of features involved, and performance declined with the addition of five TME descriptors (mMCC of 0.145), or a combination of five TME descriptors, 9 immune response scores and the EaSIeR score (mMCC of 0.105). The lowest performance (mMCC of 0.097) was observed when CART was based solely on a combination of five TME descriptors, nine immune response scores and the EaSIeR score.

Overall, integrating five TME descriptors, nine immune response scores, and the EaSIeR score, as computed using the EaSIeR software, and using these features in model training provides valuable information that can enhance predictive performance. Specifically, nine immune response scores contributed significantly to improving the accuracy of both LGBM and CART models. However, the inclusion of five TME descriptors, either alone or in combination with other features, did not consistently lead to performance gains and, in some cases, introduced additional complexity that reduced model effectiveness.

### Comparing the best-performing models with those from Automated ML (AutoML)

AutoML is the process of applying ML models to real-world problems using automation. JADBio^58^ is a powerful AutoML tool tailored to low-sample high-dimensional datasets as those arising in biomedical or biological problems. JADBio outputs optimal predictive models by automatically training and evaluating numerous ML pipelines, optimising data preprocessing, feature selection, ML algorithms and their hyperparameters. We applied JADBio on two sets of features: gene expression profiles (GEP) and integrated gene expression profiles and clinical data (GEP + clinical) to predict atezolizumab response in 320 mUC patients from merged discovery and validation datasets using regression models and compare them to our best-performing models built in this study on the same set of patients.

When using gene expression profiles as an input, JADBio performs a repeated 10-fold CV (max. repeats = 20) with 4276 configurations tried. The best-performing model is RF (with the following hyperparameters: training 100 trees with mean squared error (MSE) splitting criterion, minimum leaf size = 9, splits = 1, alpha = 1 and variables to split = number of variables divided by 9.0), along with SES feature selection (with maxK = 2, alpha = 0.1 and budget = 3 * number of variables) with MCC of 0.179, producing 6 equivalent signatures of 25 out of 19,440 genes (Table 3).

**Table 3:** The predictive performance of the best-performing models vs JADBio models in predicting atezolizumab response of 320 mUC patients from the merged discovery and validation datasets

Features	The best algorithm	Methods	#features	MCC	ROC-AUC	PR-AUC
GEP	LGBM-OMC	Nested 10-fold CV	49 genes	0.252	0.685	0.860
GEP + clinical	CART-OMC	Nested 10-fold CV	63 genes + TMB + TNB	0.253	0.664	0.870
GEP	JADBio-RF-SES^a^	Repeated 10-fold CV (maximum repeats = 20)	25 genes	0.179	0.625	0.842
GEP + clinical	JADBio-RF-SES^b^	Repeated 10-fold CV (maximum repeats = 20)	15 gene + TMB + TNB	0.198	0.660	0.834

^a^JADBio optimal parameters for GEP: RF (with hyper-parameters: training 100 trees with mean squared error (MSE) splitting criterion, minimum leaf size = 9, splits = 1, alpha = 1, and variables to split = number of variables divided by 9.0) with SES feature selection (with hyper-parameters: maxK = 2, alpha = 0.1 and budget = 3 * number of variables).

^b^JADBio optimal parameters for GEP + clinical: RF (with hyper-parameters: training 100 trees with MSE splitting criterion, minimum leaf size = 7, splits = 1, alpha = 1, and variables to split = number of variables divided by 9.0) with SES feature selection (with hyper-parameters: maxK = 2, alpha = 0.1 and budget = 3 * number of variables).

Two sets of features were used: gene expression profiles (GEP) and integrated gene expression profiles with clinical data (GEP + clinical). Out-of-sample CV predictions for the patients from JADBio’s best-performing model were obtained. To perform a direct comparison, evaluation metrics were calculated with the same script and approach from the out-of-sample CV predictions of each method. Random-level performance is 0.0 for MCC, 0.5 for ROC-AUC, 0.765 for PR-AUC when using GEP, and 0.733 for PR-AUC when using GEP + clinical.

Combining gene expression profiles and clinical data slightly improved the model performance in predicting atezolizumab response using JADBio. The best-performing model is RF (with training 100 trees with MSE splitting criterion, minimum leaf size = 7, splits = 1, alpha = 1, and variables to split = number of variables divided by 9.0), along with SES feature selection (with maxK = 2, alpha = 0.1 and budget = 3 * number of variables) with MCC of 0.198, producing 56 equivalent signatures of 19 (15 genes, TMB per megabase, and TNB per megabase) out of 19,448 features.

The best-performing models on the merged dataset, with MCC of 0.252 for GEP (LGBM-OMC) and MCC of 0.253 for GEP + clinical (CART-OMC), outperformed the best-performing JADBio models with MCC of 0.179 for GEP and MCC of 0.198 for GEP + clinical (Table 3).

## Discussion

Atezolizumab is an effective immunotherapy for treating various types of cancer^8–12^ However, only a minority of patients receiving atezolizumab benefit from it^2^. Considering the substantial risk of harmful side effects and the high cost of the treatment, it is crucial to identify predictive biomarkers to treat only those patients who are likely to benefit from this drug. In recent years, TMB, TNB and PD-L1 protein expression have been identified as potential predictors of response to immunotherapy in clinical practice^19^. However, due to the complexity of immune response and tumour biology, a single biomarker is unlikely to be sufficient to predict clinical outcomes in response to immunotherapy. Rather, the integration of multiple biomarkers may be necessary for better prediction of clinical benefit. With the advances in high-throughput sequencing technology, ML has been recently used as a promising approach that can efficiently improve the accuracy of predictive drug response models and tailor treatment for individual cancer patients^30–32,59,60^.

Based on gene expression profiles and clinical data from two independent cohorts with mUC patients receiving atezolizumab^2,41^, we demonstrated the use of regression algorithms for the classification task by applying a threshold to classify the predicted values into class labels (responder or non-responder). This approach allows the model to learn from all the patients’ response labels (C.R., P.R., S.D. and P.D.) rather than the more common information-wasting binary classes (C.R. and P.R. as responders and S.D. and P.D. as non-responders). By conducting five 10-fold CV runs on the discovery dataset to select the best OMC model, which was subsequently tested on the validation dataset, this study identified a robust ML model (CART-OMC) with the highest MCC of 0.437 for predicting atezolizumab response in mUC patients based on their gene expression profiles. The OMC model, trained only on a small subset of informative features, facilitates model prediction when dealing with high-dimensional datasets. CART-OMC nonlinearly combined only 29 (0.15%) out of the 19,441 genes considered. Thus, in a prospective clinical trial, each mUC patient would only need to be profiled for these 29 genes before treatment, which would represent a large saving in time and cost with respect to determining the full profile. Additionally, combining gene expression profiles and clinical data, GEP + TMB + TNB (LR-OMC), did not improve predictive accuracy. This lack of improvement could be attributed to heterogeneity in TMB and TNB detection methodologies and quantification across different laboratories, which may reduce the predictive performance of models incorporating TMB and TNB into their predictions.

The predictive performance of CART-OMC is robust in this context. First, CART-OMC demonstrated greater predictive power than TMB, TNB, and PD-L1 protein expression, which have been identified as immunotherapy response biomarkers (Fig. 3). Second, after clustering 374 mUC patients of the discovery (*N* = 348) and validation datasets (*N* = 26) with known and unknown atezolizumab response records based on the 29 predictive genes from CART-OMC (Fig. S1), 298 atezolizumab-treated mUC patients in the training dataset were well-represented in all the clusters into which the 26 mUC patients in the validation dataset and other patients with unknown response records were partitioned. This suggests that these patients fall within the applicability domain of the model, and thus are expected to have similar accuracy when predicting their response to atezolizumab. Third, CART-OMC could identify 29 genes that are potentially associated with atezolizumab response and its mechanism. Wikicancer and Go pathway enrichment analysis of these 29 genes revealed that a subset was significantly enriched (FDR < 0.05) in 32 biological pathways related to cancer-associated and immunotherapy response mechanisms (Fig. S6, Table S6). These 29 genes might be involved in atezolizumab resistance and cancer progression through alterations in their expression.

Lastly, we merged the discovery and validation datasets to obtain a more challenging test set. With a total of 320 mUC patients, we were able to generate predictions for those patients and compare our model’s performance with state-of-the-art ML drug response prediction models (e.g. EaSIeR and JADBio). Our best-performing models (LGBM-OMC with 49 genes, and CART with 63 genes with TMB and TNB) outperformed EaSIeR (Fig. 4) and JADBio (Table 3) in predicting response to atezolizumab in 320 mUC patients, despite implementing a fully autoML approach and extensive tuning effort in JADBio. Furthermore, integrating predictive genes from the best-performing models with five TME descriptors, nine published immune response scores, and the EaSIeR score, as computed for each patient using EaSIeR, improved the prediction of atezolizumab response (Fig. 5). This integration provides a comprehensive approach that incorporates various dimensions of tumour-immune interactions, leading to improved predictive accuracy. Despite merging more features computed using EaSIeR providing a performance boost, we should consider the time and cost overhead of determining the full gene expression profiles in every patient for EaSIeR RNA-sequencing input to compute these features.

Due to the challenging problem in this study associated with predicting atezolizumab response in mUC patients, the EaSIeR model employed a multi-task ML built on TCGA cancer patients who were not treated with anti-PD-1/anti PD-L1 inhibitors. Despite benefiting from the availability of many samples, a major limitation is that the model may not accurately predict atezolizumab response from non-immunotherapy-treated TCGA patients. Unlike our approach, which involves training the models on gene expression profiles and clinical data from mUC patients treated with atezolizumab. Additionally, JADBio has a limited number of algorithms to build the model. Thus, it may need to consider including additional algorithms such as LGBM, which was the best-performing model in our study, to handle a wider range of datasets and achieve higher accuracy in predictions.

Developing a predictive model for precision oncology is a challenging task that requires collecting and analysing a significant amount of data. Although the best-performing models have shown promising results, there are some limitations to be considered in this study. Firstly, it was only an in silico and retrospective analysis of publicly available data from the IMvigor210 clinical trial and Snyder et al. studies^2,41^, with a limited number of mUC patients treated with atezolizumab and lack relevant clinical variables such as tumour stage at diagnosis and metastatic sites, making it challenging to apply the model in a context other than metastatic settings. A curated larger multi-cohort dataset beyond these sources should be further used to build a model with enhanced predictive accuracy and reliability across different disease stages and patient populations, making it more useful for guiding treatment decisions. In addition, external validation on publicly available datasets or prospective validation in clinical studies could be considered to further establish its robustness and generalisability.

Notably, our previous work has also demonstrated the feasibility of applying ML models to predict drug responses in both early-stage and metastatic cancer patients treated in the adjuvant setting following surgery, for example, doxorubicin in breast cancer patients^31^, gemcitabine in pancreatic cancer patients^32^, as well as cetuximab in CRC PDXs^29^. These studies show that ML models can be adapted to such scenarios, providing machine learning models able to discriminate between sensitive and resistant cases, thereby providing a foundation for future work exploring this potential. Secondly, the effect of drug combinations could be further evaluated along with their individual effects. Another limitation is that incorporating other modalities, such as imaging or single-cell profiling, could help develop a predictive model. This can provide insight into the heterogeneity of the tumour, leading to a more comprehensive understanding of the disease, and potentially improve treatment decisions.

Despite the evolving treatment landscape for metastatic urothelial carcinoma (mUC)^61^, atezolizumab remains a clinically relevant option, particularly for patients ineligible for platinum-based chemotherapy. It is also used in cisplatin-eligible patients with PD-L1+ tumours (≥5% immune cell expression) and in more advanced treatment settings^62^. Future work could also adapt our methodology to develop and evaluate an ML pipeline for predicting patient responses to other ICIs, such as pembrolizumab, thereby broadening the model’s predictive scope beyond atezolizumab-based treatments. Incorporating data from multiple ICIs and investigating the molecular signatures of response could lead to a more generalised predictive model, applicable across a wider range of immunotherapies, including emerging combination regimens like enfortumab vedotin (EV) plus pembrolizumab. In conclusion, this ML analysis has led to the discovery of highly predictive and robust predictors of mUC patient response to atezolizumab based on gene expression profiles, which achieved MCC values that are higher than those based on a single biomarker (e.g. TMB, TNB and PD-L1 protein expression alone), and other ML-based prediction models from EaSIeR and JADBio. The predictive features identified through this study can serve as response signatures for atezolizumab. This enables clinicians to stratify mUC patients who will likely respond to atezolizumab and provide alternative drugs without delay for those who are unlikely to respond, thus minimising the incidence of adverse effects. Furthermore, these findings provide a starting point for investigating the mechanism of atezolizumab resistance in mUC patients.

## Methods

### Gene expression profiles and clinical data acquisition

We retrospectively collected two publicly available datasets for model development and validation. The IMVigor210 phase II clinical trial of atezolizumab (1200 mg intravenous every 3 weeks) in mUC patients was used as the discovery dataset for model development^2^. We retrieved the RNA-sequencing data, along with clinical features, through the IMvigor210CoreBiologies R package (http://research-pub.gene.com/IMvigor210CoreBiologies/). For the validation dataset, we used mUC patients receiving atezolizumab (1200 mg intravenous every 3 weeks) published by Snyder et al.^41^. The RNA-sequencing data and clinical features were available at 10.5281/zenodo.546110. We included only the patients with gene expression profiles and known atezolizumab response annotated, omitting individuals missing these data. Thus, 298 and 22 gene expression profiles of atezolizumab-treated mUC patients remained for the discovery and validation datasets, respectively. Table 1 summarises the patients’ characteristics that we used in this study. Atezolizumab response was evaluated by response evaluation criteria in solid tumours (RECIST) version 1.1 based on computed tomography (CT) or magnetic resonance imaging (MRI) scans^63^. Patients who achieved complete response (CR) or partial response (PR) were defined as responders, while patients with stable disease (SD) or progressive disease (PD) were defined as non-responders. Thus, the response rate was 23% (68 out of 298 patients) in the discovery dataset and 32% (7 out of 22 patients) in the validation dataset.

### Data preparation for ML

Our computational framework, illustrated in Fig. 1, outlines the process to develop and validate the predictive ability of gene expression profiles on mUC patients’ response to atezolizumab. We identified 19,441 genes that overlapped between the discovery and validation datasets and transformed them as log2(count + 1) for further analysis. The patients’ response labels were mapped to 1.0, 2.0, 3.0 and 4.0 for CR, PR, SD, and PD, respectively. The discovery dataset was randomly split into *K* subsets using *K*-fold (for three *K* values: 5, 10 and the number of samples in the dataset). Eight regression algorithms, including classification and regression tree (CART), random forest (RF), extreme gradient boosting (XGB), light gradient boosting machine (LGBM), K-nearest neighbours (KNN) regression, linear regression (LR), support vector machine (SVM) with linear kernel and SVM with the radius basis function kernel (RBF), employed OMC feature selection^29^, were used to predict patients’ atezolizumab response. The standard 10-fold cross-validation (CV) was performed five times, each time with a different random seed, to assess the robustness of the ML model. All analyses were performed using the Python package version 3.7.3 (https://www.python.org/) with packages from scikit-learn version 0.24.2 (https://scikit-learn.or).

### Building OMC regression models

The OMC models were implemented to mitigate the impact of the high dimensionality of the data; in this case, the number of features is much larger than the number of patients. OMC builds a model using only a small subset of informative features while discarding irrelevant ones to facilitate model prediction. The standard *K*-fold CV was used to estimate the predictive performance of each algorithm using only the most relevant features to atezolizumab responses. In brief, the *P*-value of each feature was calculated using the analysis of variance (ANOVA) test, with a small *P*-value indicating high discriminatory power to distinguish atezolizumab response. Then, each of 8 regression algorithms was trained on *K* − 1 folds using only the considered subsets of features (the top 2 to *n*/2 subset of features, where *n* is the number of samples), and tested on the remaining one fold for testing the model. The CV was then repeated *K* times, with each of the *K* folds being used exactly once as the test set. Thus, each patient has exactly one out-of-sample prediction. The resulting out-of-sample predictions were merged from all folds, and the Matthews correlation coefficient (MCC) was calculated once with all samples. Among all *n*/2 trained models, the best OMC model, the one with the highest MCC, was trained on all samples of the discovery dataset and subsequently used to predict samples in the validation dataset.

### Building models based on immunotherapy biomarkers

To compare the performance of the best-performing model with that of individual immunotherapy biomarkers: TMB per megabase, TNB per megabase, or PD-L1 protein expression status on TICs by immunohistochemistry assay (IC0: <1%, IC1: ≥1% but <5% and IC2: ≥5%) as provided by the original study. We trained each feature with the LR algorithm and evaluated it with a standard 10-fold CV. The merged out-of-sample predictions from the 10 folds were used to calculate model performance metrics.

### Model performance evaluation

Atezolizumab responses were encoded as follows: CR as 1, PR as 2, SD as 3 and PD as 4. We defined a responder as a patient who had CR or PR, and a non-responder as a patient who had SD or PD. The merged out-of-sample predictions from *K*-folds were categorised into binary classes using a threshold of 2.0, since CR and PR were considered as responders. Patients with predicted responses exceeding the threshold were classified as non-responders; otherwise, they were classified as responders. Then, the four confusion matrix categories, including true positive (TP), true negative (TN), false positive (FP) and false negative (FN), were counted and used to calculate evaluation metrics, including MCC, ROC-AUC and the area under the precision-recall curve (PR-AUC).

### Prediction of response to atezolizumab using the EaSIeR

EaSIeR^29^ is an R Bioconductor package developed to assess patients’ response to anti-PD-1/anti PD-L1 inhibitors from bulk RNA sequencing of their tumours. EaSIeR quantifies patient-specific, real-valued five types of system-based signatures of TME, including 11 immune cell types, 14 pathway activities, 118 transcriptional factor activities, 812 ligand–receptor pairs and 169 cell–cell pairs. The cancer-specific elastic net regularised multi-task linear regression (RMTLR) model, built on TCGA samples, was used to predict 10 tasks of immune response scores: cytolytic activity (CYT), Ock immune signature (Ock_IS), Roh immune score (Roh_IS), Chemokine signature (Chemokine), Davoli immune signature (Davoli_IS), IFNy signature (IFNy), expanded immune signature (Ayer_expIS), T-cell inflamed signature (Tcell_inflamed), repressed immune resistance (RIR), tertiary lymphoid structure signature (TLS) based on individuals or a combination of five system-based signatures of TME. These ten immune response scores, which have been experimentally validated in the literature (Table S1) comprehend key gene signatures for immune activation in the TME in relevant contexts. They reflect patients’ likelihood of responding to ICI inhibitors, with higher scores indicating a higher likelihood of responding to these therapies.

We did not train the EaSIeR model on the discovery dataset and evaluate it on the validation dataset, as done with our method, because EaSIeR cannot be retrained on new datasets. Instead, the bladder urothelial carcinoma (BLCA)-specific RMTLR model, pre-trained on TCGA-BLCA samples available in the EaSIeR package, was used to predict the likelihood of response to atezolizumab in 320 mUC patients (75 responders and 245 non-responders) from the merged discovery and validation datasets. The model generated 60 EaSIeR predictions by computing 10 immune response scores from six sets of features, which included five system-based TME signatures and their combinations.

To compare predictive performance in this task, we developed OMC models using the same set of 320 mUC patients from the merged discovery and validation datasets. Two feature sets were used for each learning algorithm: gene expression profiles alone, and integrated gene expression profiles with clinical data. Each model was evaluated using a nested 10-fold CV (the CV is repeated five times changing random seed).

To calculate predictive performance, the model’s predictions from both our approach and the EaSIeR were normalised to a range of 0–1 in order to allow for fair comparison and evaluation across different models, regardless of their scale of prediction outputs. Class labels (responder or non-responder) were assigned based on a threshold of 0.5 on the normalised predicted values, and evaluation metrics were then calculated.

### Prediction of response to atezolizumab using the JADBio

Just Add Data Bio (JADBio) version 1.4.94 (www.jadbio.com)^58^ is an automated ML (AutoML) platform used to develop regression models for predicting atezolizumab response in 320 mUC patients from the merged discovery and validation datasets. Two sets of features were considered: gene expression profiles alone and gene expression profiles integrated with clinical data. To identify the best-performing model, JADBio applied several regression algorithms for modelling, including ridge linear regression, support vector regression with linear and Gaussian kernels, random forest, and decision tree. Feature selection was performed using the least absolute shrinkage and selection operator (LASSO) and Statistically Equivalent Signature (SES) algorithms, while various hyperparameter values (Table S2) were evaluated to optimise the model’s performance. JADBio carried out a repeated 10-fold CV (maximum repeats = 20) to evaluate the model performance. The “extensive tuning effort” option was selected to extensively search for an optimal model at the expense of computational cost. The best-performing model predictions were saved, and evaluation metrics were calculated using normalised out-of-sample predictions (ranging from 0 to 1) from the CV runs in JADBio, following the same way as our approach for comparison purposes. Class labels (responder or non-responder) were assigned based on a threshold of 0.5.

### Statistical analysis

The two-tailed unpaired Welch’s *t*-test was used to generate *P*-values in data analysis, with a *P*-value less than 0.05 considered statistically significant. For pathway enrichment analysis, WebGestalt uses a hypergeometric test for statistical significance, which further employs the Benjamini–Hochberg multiple-testing correction for the correct *P*-value. The significantly enriched pathway was identified with a false discovery rate (FDR) less than 0.05.

## Supplementary information

Supplementary Information

## Data Availability

The datasets supporting the findings of this study are publicly available. The RNA-sequencing data and associated clinical features for the IMvigor210 discovery cohort were obtained through the IMvigor210CoreBiologies R package (http://research-pub.gene.com/IMvigor210CoreBiologies/). The Snyder validation cohort is available at Zenodo (10.5281/zenodo.546110). All Python code and the processed gene expression dataset used to build and evaluate the best-performing model (CART-OMC) for predicting atezolizumab response in metastatic urothelial carcinoma (mUC) patients are available at 10.5281/zenodo.15162660. This resource is intended to support reproducibility and facilitate application to other gene expression-profiled mUC cohorts.
