# Supplementary material for: Predicting atezolizumab response in metastatic urothelial carcinoma patients using machine learning on integrated tumour gene expression and clinical data
Source: NPJ Precis Oncol. 2025 Jun 10;9:170. doi: 10.1038/s41698-025-00969-8 (PMC12152156; doi:10.1038/s41698-025-00969-8)
Supplement: Supplementary file 1 — Supplementary Information [file 41698_2025_969_MOESM1_ESM.docx]

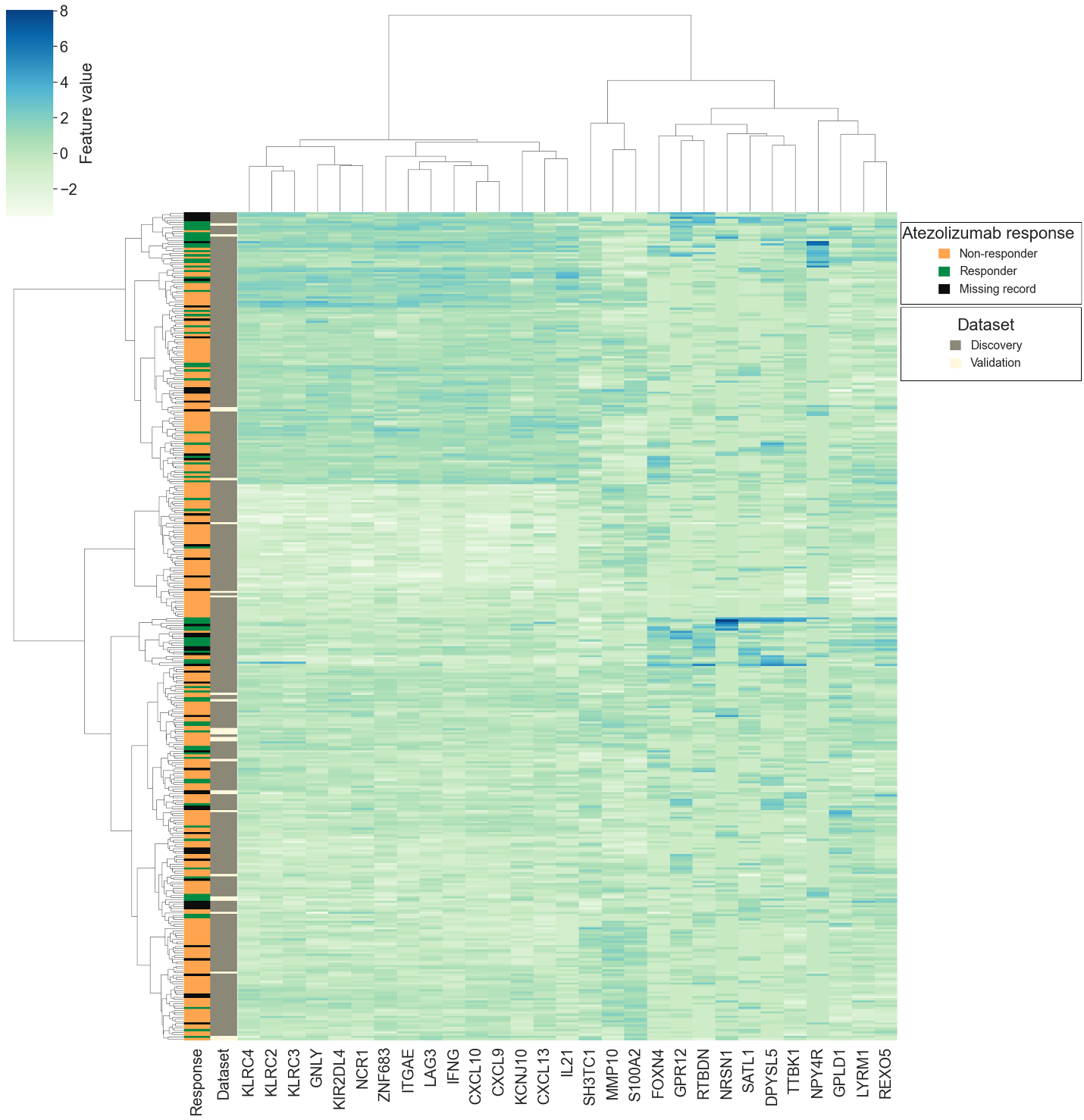


**Figure S1. Unsupervised clustering heatmap of 29 predictive genes in the atezolizumab-treated mUC patients.** The normalised log2-transformed gene expression of 29 predictive genes from the GEP (CART-OMC) model was used to cluster 374 mUC patients of the discovery (N = 348) and validation datasets (N = 26). On the left, colour bars represent atezolizumab responders (green), non-responders (orange), and patients with unknown response records (black). Patients from the discovery or validation datasets were coloured grey and yellow, respectively. The colours on the top-left of the heatmap represent the normalised expression of the 29 predictive genes. The horizontal axis represents predictive features, and the vertical axis represents patients. Both the rows of patients and the columns of genes have been clustered, with similar patients and genes grouped together.


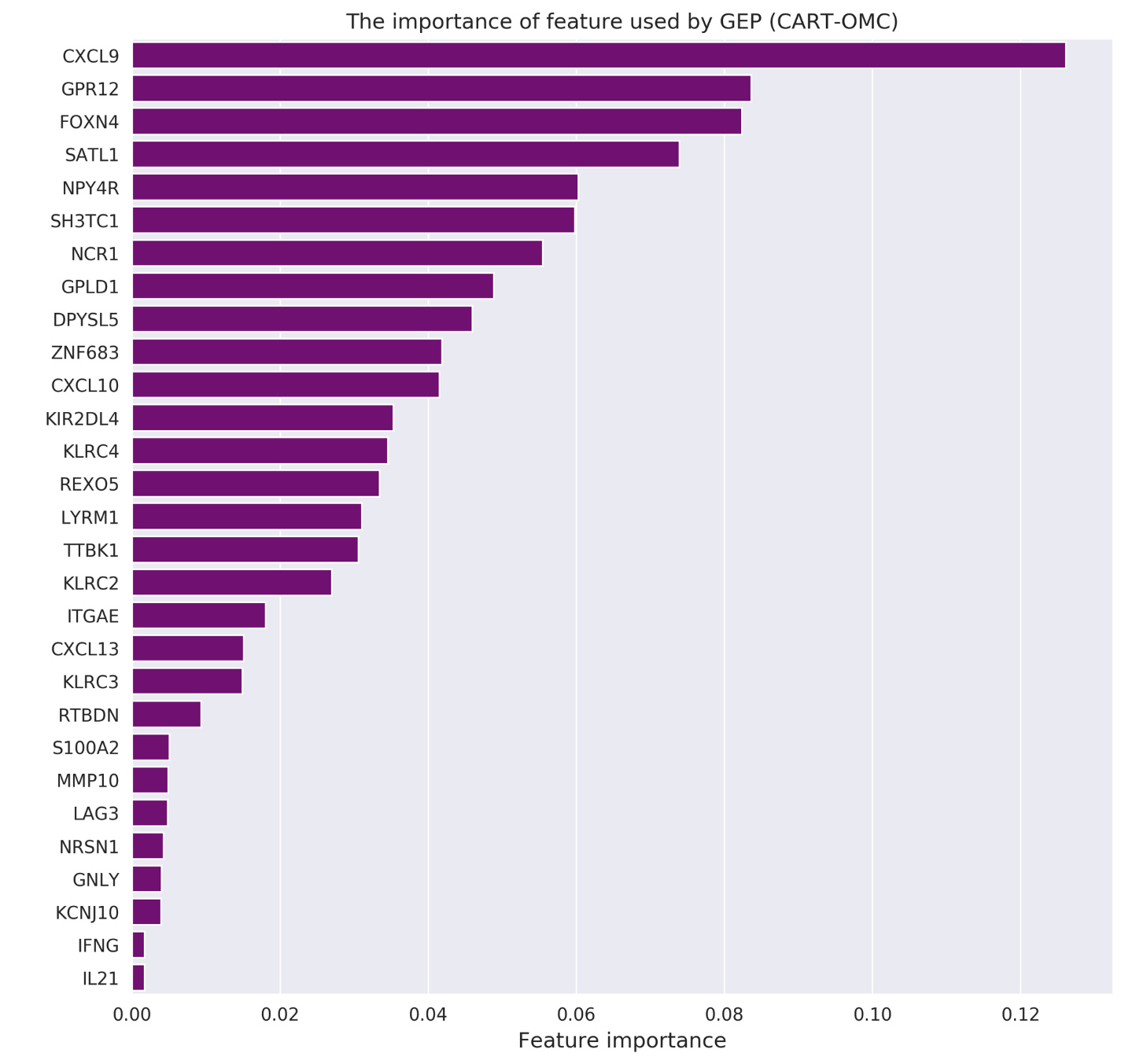


**Figure S2**. **Feature importance plot of 29 predictive genes from the most predictive model (CART-OMC) for predicting atezolizumab responses in mUC patients.** The 29 important features are listed and sorted by the feature importance score, computed as the reduction of Gini impurity by each feature of the decision tree. The reduction in the important scores means less contribution to the predictive model for predicting atezolizumab responses in mUC patients. CXCL9 is the most important feature used by the CART-OMC model.


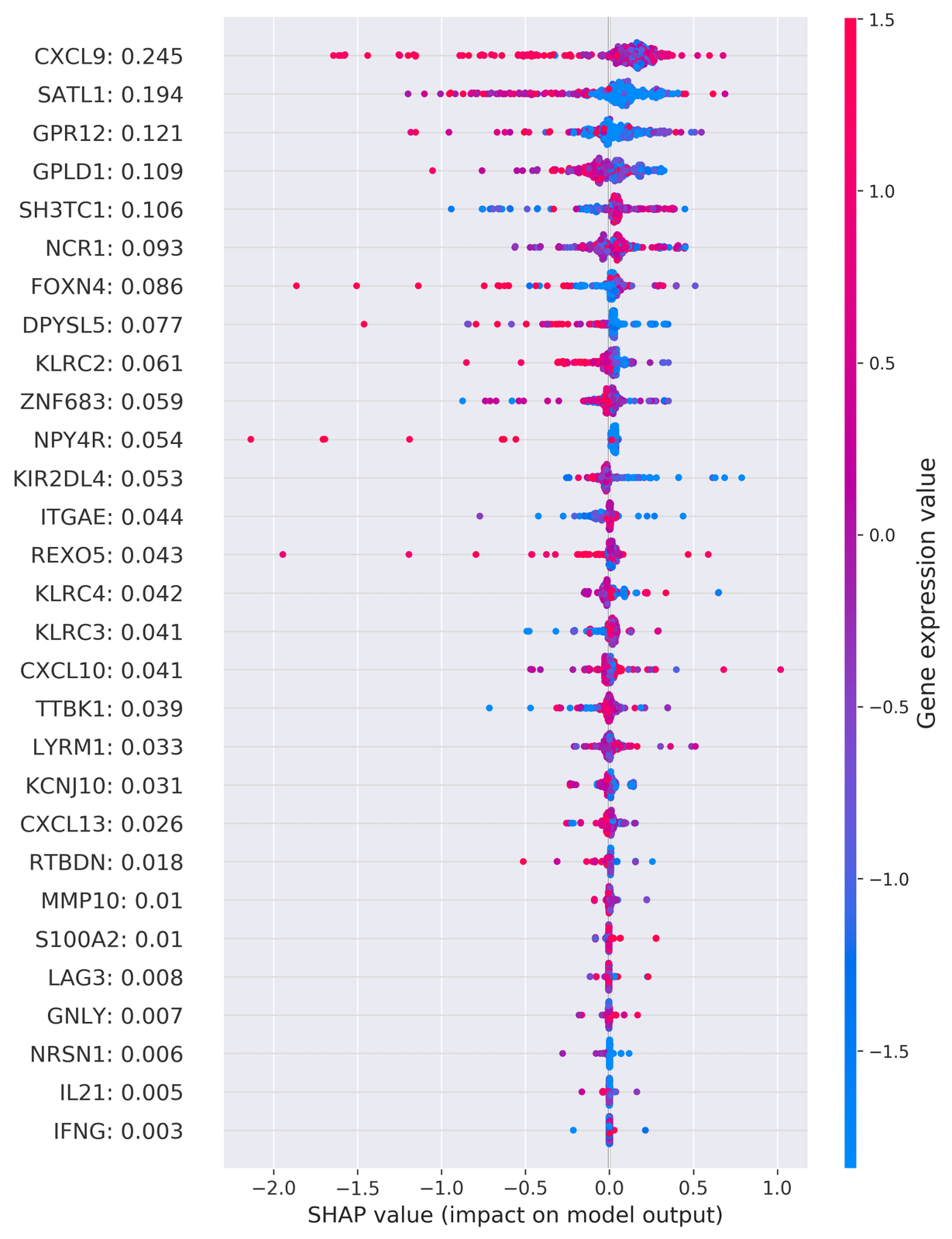


**Figure S3. Model interpretation using SHAP (Shapley Additive exPlanations).** The SHAP summary plot depicts the importance of 29 predictive genes from CART-OMC in predicting 320 mUC patients from the merged discovery and validation datasets (75 responders and 245 non-responders). The 29 genes are listed and sorted according to averaged absolute SHAP values across all patients (the value immediately to the right of the gene name), with larger values indicating higher importance. Each horizontal line of dots represents gene expression levels for individual patients, with colours ranging from low (blue) to high (red). The x-axis shows SHAP values for individual patients. The further away from the vertical line at x = 0, the larger the impact on the output prediction. A negative SHAP value to the left increased the chance of being a responder, while a positive SHAP value to the right increased the chance of being a non-responder. CXCL9 is the most important feature in the model’s prediction, with higher expressions associated with predicted responders.


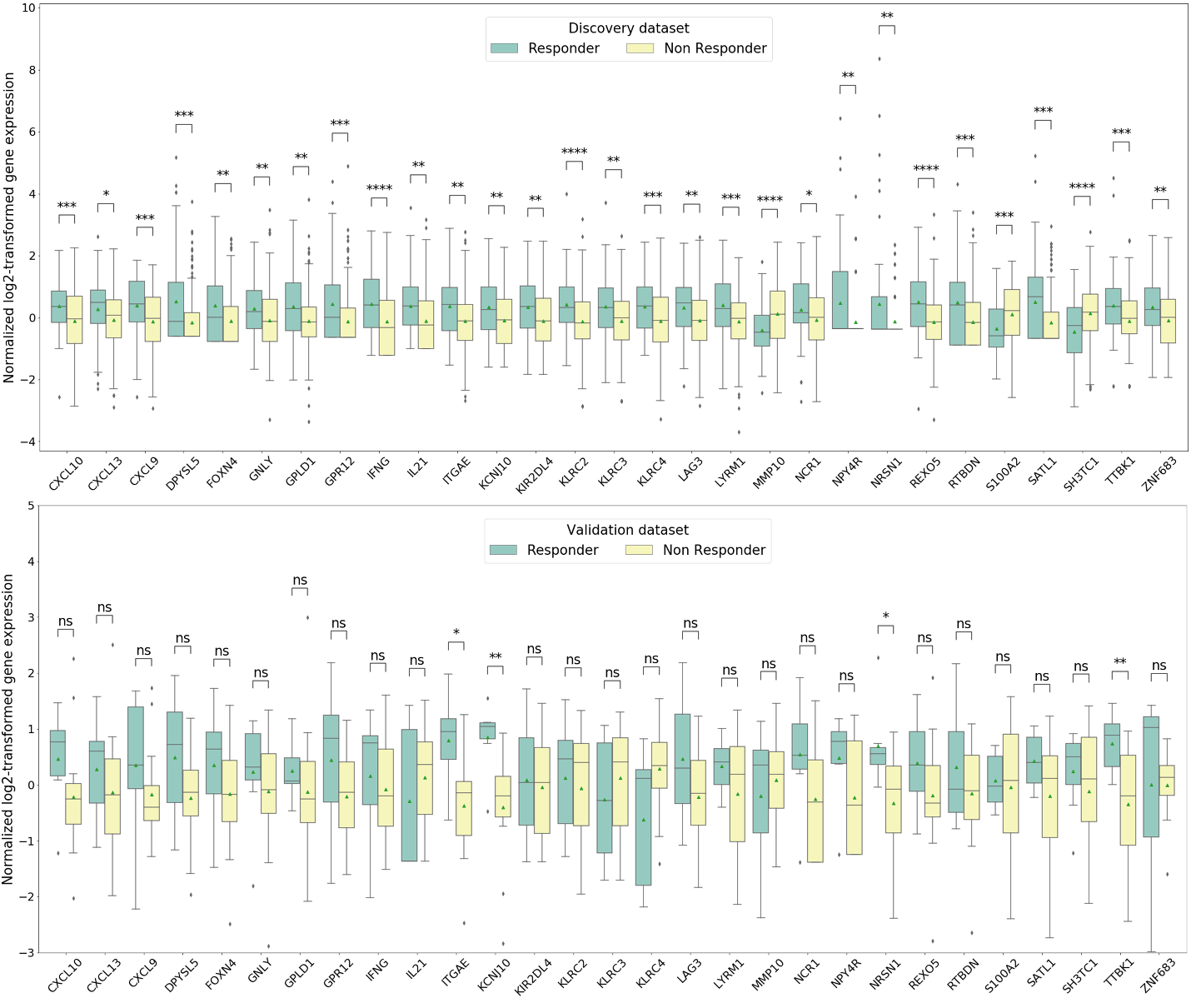


**Figure S4. Boxplot showing the expression pattern of the 29 predictive genes from the best-performing model (CART-OMC) between responders and non-responders.** The y-axis represents the normalized log2-transformed gene expression of 29 genes obtained from the GEP (CART-OMC) model between responders and non-responders in the discovery (top) and validation (bottom) datasets. The line inside each boxplot represents the median expression across patients for a given feature, while the green triangle represents the mean expression. The box plot for each feature contains the *P*-value of the mean differential expression between responders and non-responders using a two-sided Welch’s t-test. Stars denote the p-value, where non-significant “ns” means 0.05 < p ≤ 1.00, “*” means 0.01 < p ≤ 0.05, “**” means 0.001< p ≤ 0.01, “***” means 0.0001 < p ≤ 0.001, and “****” means p ≤ 0.0001. A p-value less than 0.05 is considered as a significant difference in expression levels between the two groups.


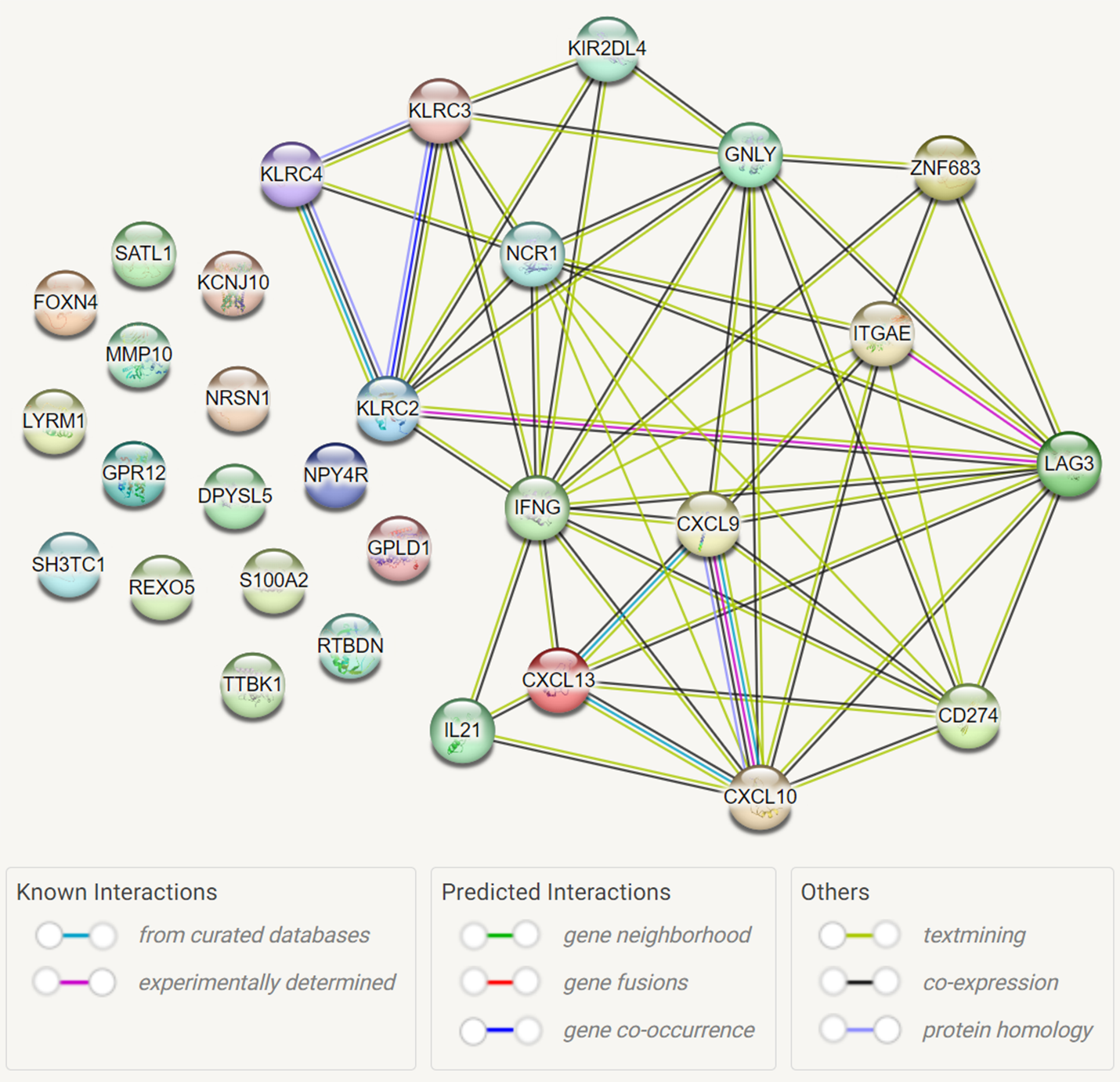


**Figure S5. The protein-protein interaction (PPI) network analysis.** 29 predictive genes identified from the best-performing model (CART-OMC), along with the CD274 gene coding for PD-L1 (atezolizumab’s primary target), were input into the STRING database (https://string-db.org/) for PPI network analysis. We limited the species to “Homo sapiens” and required at least the minimum confidence interaction score > 0.4. As a result, the proteins were connected as a group with 30 nodes and 54 edges. The nodes in the PPI network represent protein-coding genes, and the edges represent their known and predicted interactions, as shown in the legend. A PPI enrichment p-value of 0.0001 was obtained, indicating that the protein input has more interactions among themselves than what would be expected for a set of proteins of the same size and degree distribution drawn at random from the genome.


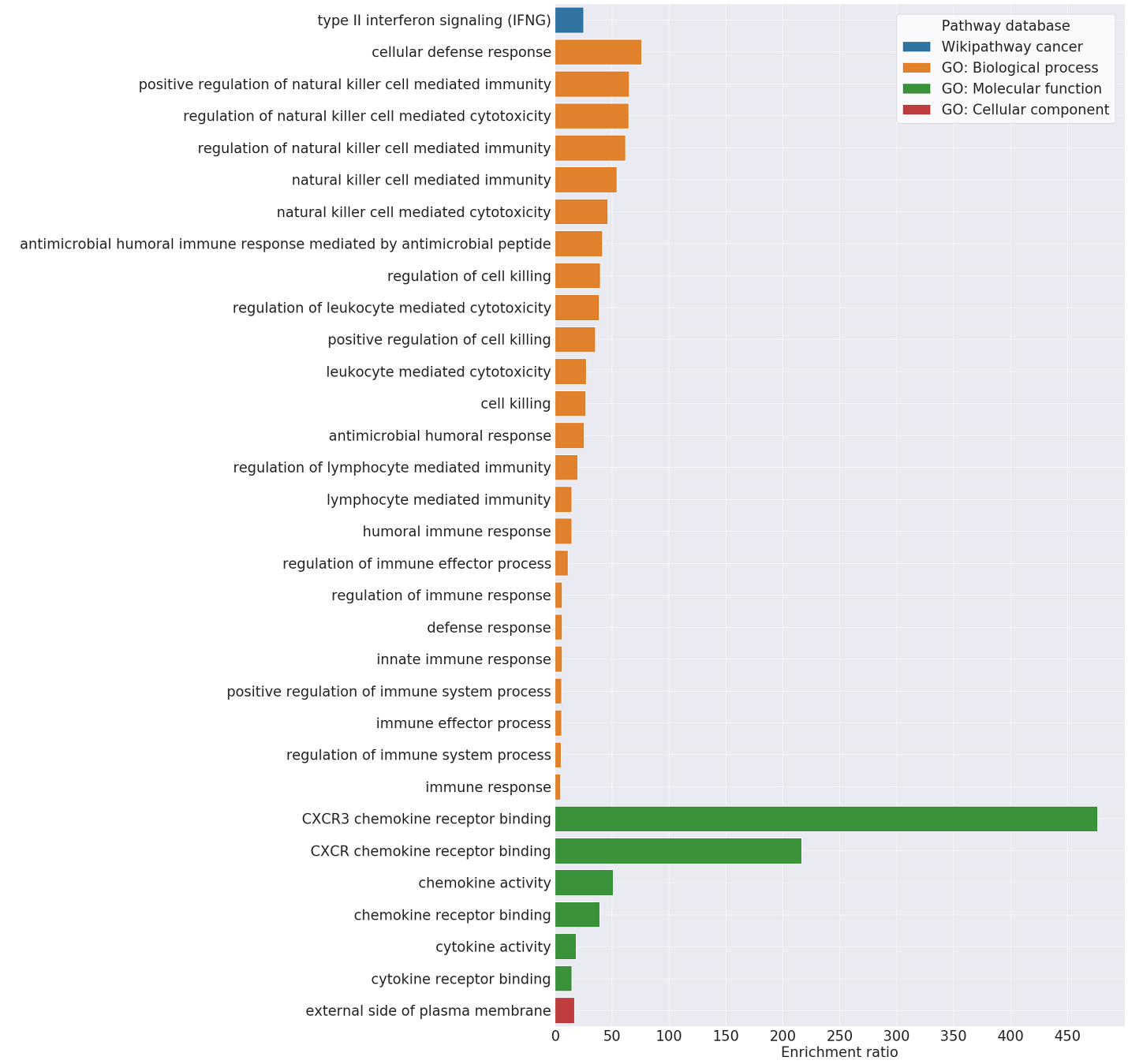


**Figure S6. Wikipathway cancer and Gene Ontology (GO) pathway enrichment analysis of 29 predictive genes for predicting atezolizumab response in mUC patients.** The pathway enrichment analysis was performed using the cancer-related repository of WikiPathways (WikiPathway cancer) and the GO database in WebGestalt. The bar chart summarizes 32 biological pathways that are significantly (FDR ≤ 0.5) enriched with different subsets of 29 genes in the Wikipathway cancer (blue) and the GO terms, including biological process (orange), molecular function (green), cellular component (red), and presented on the y-axis. The x-axis represents the enrichment ratio, calculated as the number of genes overlaps with the reference gene set in a given pathway. OMC highlights genes known to play a role in the mechanism of the drug when applied to mUC. Our best-performing model (CART-OMC) could identify 29 genes that are primarily cancer-related and potentially associated with atezolizumab response and its mechanism, including cellular defense response, immune cell-mediated immunity and cytotoxicity, cell killing, and regulation of immune system process and immune response, chemokine and cytokine receptor binding pathways.


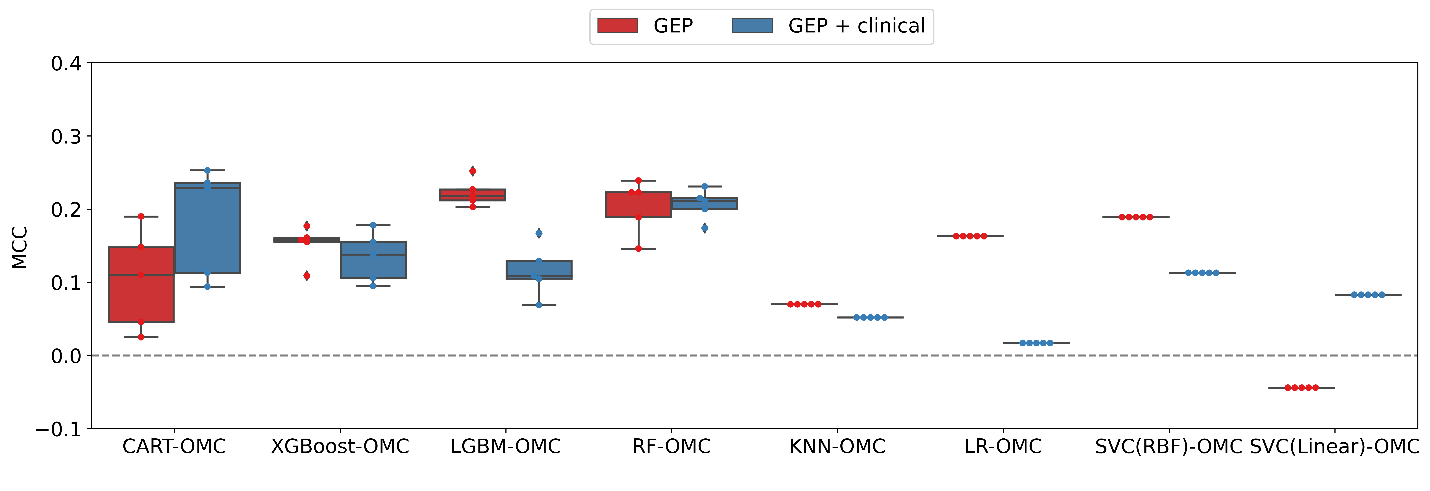


**Figure S7.** **Five-repetition MCCs on the merged discovery and validation datasets across eight ML models using either gene expression profiles or integrated gene expression profiles plus clinical data.** MCC is calculated using a merged prediction of a nested 10-fold CV run on the merged discovery and validation datasets (245 non-responders and 75 responders). The CV is repeated five times with different random seeds to assess robustness, and the results are presented in a boxplot. Eight regression algorithms using OMC feature selection to select only a small subset of informative features facilitating model prediction. Two sets of features were employed per learning algorithm: gene expression profiles (GEP) and integrated gene expression profiles with clinical data (GEP + clinical). An MCC of 0.0 indicates random-level performance, as shown by the horizontal dashed line.

**Table S1.** presents 10 published signatures of the immune response.

| **No.** | **Published signatures of immune response** | **Study** | **Explanation** |
| --- | --- | --- | --- |
| 1. | Cytolytic activity (**CYT**) | Rooney et al., 2015 | The level of two cytolytic effectors, granzyme A and perforin, which are overexpressed upon CD8+ T cell activation |
| 2. | Ock immune signature (**Ock_IS**) | Ock et al., 2017 | The expression of 105 genes associated with the response to immunotherapy with the MAGE-A3 antigen |
| 3. | Roh immune score (**Roh_IS**) | Roh et al., 2017 | A set of genes involved in immune activation in relation to tumor rejection |
| 4. | Chemokine signature (**chemokine**) | Messina et al., 2012 | A gene set associated with inflammation and immunity, which is able to predict host immune reaction and the formation of tumor-localized lymphoid structure. |
| 5. | Davoli immune signature (**Davoli_IS**) | Davoli et al., 2017 | The expression of cytotoxic CD8+ T cell and NK cell markers |
| 6. | IFNy signature (**IFNy**) | McClanahan et al., 2017 | Genes able to separate responders and non-responders in melanoma |
| 7. | Expanded immune signature (**Ayer_expIS**) | McClanahan et al., 2017 | Genes highly correlated with IFN-γ signature genes; this new set included all immune-related genes. |
| 8. | T-cell inflamed signature (**Tcell_inflamed**) | McClanahan et al., 2017 | The joint potential of IFN-γ and T-cell-associated inflammatory genes in predicting response to PD-1 blockade |
| 9. | Repressed immune resistance (**RIR**) | Jerby-Arnon et al., 2018 | Combining a set of gene signatures associated with T cell exclusion, post-treatment, and functional resistance. |
| 10. | Tertiary lymphoid structure signature (**TLS**) | Cabrita et al., 2020 | Differentially expressed genes in tumors with TLS |

**Table S2.** The algorithms, feature selection methods, and a range of hyperparameter values tried in the JADBio

| **Feature selection** | | | |
| --- | --- | --- | --- |
| **Algorithms** | **Hyperparameters** | **min** | **max** |
| SES | maxK | 2 | 3 |
|  | alpha | 0.01 | 0.1 |
| LASSO | penalty | 0 | 2 |
| Univariate | maxVars | 100 | |
|  | alpha | 0.001 | 0.01 |
| **Modeling** | | | |
| **Algorithms** | **Hyperparameters** | **min** | **max** |
| Ridge linear regression | lamda | 0.0001 | 100 |
| Linear support vector regression | epsilon | 0.0001 | 0.5 |
|  | cost | 0.001 | 100 |
| Gaussian support vector regression | epsilon | 0.0001 | 0.5 |
|  | cost | 0.001 | 100 |
|  | gamma | 0.001 | 100 |
| Random forest | minLeafSize | 3 | 9 |
|  | nTrees | 100 | 1000 |
| Decision tree | minLeafSize | 3 | 9 |
|  | alpha | 0.01 | 0.1 |

**Table S3 (related to Figure 2).** Five-repetition evaluation metrics of the validation dataset across seven ML models using either gene expression profiles or integrated gene expression profiles plus clinical data. The best optimal model complexity (OMC) model, determined through a standard 10-fold CV on the discovery dataset, was subsequently tested on the validation dataset (the CV is performed 5 times changing the random seed). Seven regression algorithms using OMC feature selection to select only a small subset of informative features facilitate model prediction. Two sets of features were employed per learning algorithm: gene expression profiles (GEP) and integrated gene expression profiles with clinical data (GEP + clinical). Random-level performance is delimited by the horizontal dashed lines (0.0 for MCC, 0.5 for ROC-AUC, and 0.772 and 0.682 for PR-AUC when using GEP or GEP + clinical features, respectively). The best-performing model was selected based on the highest median MCC as highlighted in bold.

| **Gene expression profiles (GEP)** | | | |
| --- | --- | --- | --- |
| **Algorithms** | **Highest MCC**  **median MCC ± SD** | **Highest ROC-AUC**  **Median ROC-AUC ± SD** | **Highest PR-AUC**  **Median PR-AUC ± SD** |
| **CART-OMC** | **0.437**  **0.328 ± 0.14** | **0.881**  **0.743 ± 0.12** | **0.955**  **0.883 ± 0.07** |
| XGB-OMC | 0.463  0.319 ± 0.17 | 0.743  0.705 ± 0.08 | 0.888  0.813 ± 0.08 |
| LGBM-OMC | 0.463  0.297 ± 0.23 | 0.733  0.724 ± 0.04 | 0.879  0.861 ± 0.03 |
| RF-OMC | 0.0  0.0 ± 0.0 | 0.814  0.671 ± 0.07 | 0.907  0.843 ± 0.04 |
| LR-OMC | -0.216  -0.216 ± 0.0 | 0.457  0.457 ± 0.0 | 0.753  0.753 ± 0.0 |
| SVC(RBF) | 0.0  0.0 ± 0.0 | 0.524  0.524 ± 0.0 | 0.680  0.680 ± 0.0 |
| SVC(Linear) | 0.319  0.319 ± 0.0 | 0.705  0.705 ± 0.0 | 0.861  0.861 ± 0.0 |
|  | | | |
| **Integrated gene expression profiles with clinical data (GEP + clinical)** | | | |
| **Algorithms** | **Highest MCC**  **median MCC ± SD** | **Highest ROC-AUC**  **Median ROC-AUC ± SD** | **Highest PR-AUC**  **Median PR-AUC ± SD** |
| CART-OMC | 0.143  0.069 ± 0.09 | 0.582  0.520 ± 0.04 | 0.792  0.780 ± 0.03 |
| XGB-OMC | 0.289  0.0 ± 0.24 | 0.571  0.520 ± 0.06 | 0.758  0.705 ± 0.04 |
| LGBM-OMC | 0.289  0.115 ± 0.19 | **0.735**  **0.500 ± 0.14** | **0.867**  **0.747 ± 0.07** |
| RF-OMC | 0.289  0.289 ± 0.0 | 0.633  0.592 ± 0.03 | 0.816  0.795 ± 0.02 |
| **LR-OMC** | **0.316**  **0.316 ± 0.0** | 0.510  0.510 ± 0.0 | 0.643  0.643 ± 0.0 |
| SVC(RBF) | 0.0  0.0 ± 0.0 | 0.490  0.490 ± 0.0 | 0.650  0.650 ± 0.0 |
| SVC(Linear) | 0.115  0.0 ± 0.0 | 0.663  0.663 ± 0.0 | 0.722  0.722 ± 0.0 |

**Table S4.** The list of 69 predictive features (67 genes, TMB per megabase, and TNB per megabase) derived from the best-performing model (LR-OMC) employing integrated gene expression profiles with clinical data.

| **Entrez gene** | **Gene symbol** | **Gene name** |
| --- | --- | --- |
| 356 | FASLG | Fas ligand |
| 1521 | CTSW | cathepsin W |
| 2012 | EMP1 | epithelial membrane protein 1 |
| 2114 | ETS2 | ETS proto-oncogene 2, transcription factor |
| 2150 | F2RL1 | F2R like trypsin receptor 1 |
| 2196 | FAT2 | FAT atypical cadherin 2 |
| 2999 | GZMH | granzyme H |
| 3458 | IFNG | interferon gamma |
| 3627 | CXCL10 | C-X-C motif chemokine ligand 10 |
| 3682 | ITGAE | integrin subunit alpha E |
| 3752 | KCND3 | potassium voltage-gated channel subfamily D member 3 |
| 3766 | KCNJ10 | potassium inwardly rectifying channel subfamily J member 10 |
| 3804 | KIR2DL3 | killer cell immunoglobulin like receptor, two Ig domains and long cytoplasmic tail 3 |
| 3805 | KIR2DL4 | killer cell immunoglobulin like receptor, two Ig domains and long cytoplasmic tail 4 |
| 3811 | KIR3DL1 | killer cell immunoglobulin like receptor, three Ig domains and long cytoplasmic tail 1 |
| 3812 | KIR3DL2 | killer cell immunoglobulin like receptor, three Ig domains and long cytoplasmic tail 2 |
| 3822 | KLRC2 | killer cell lectin like receptor C2 |
| 3852 | KRT5 | keratin 5 |
| 3902 | LAG3 | lymphocyte activating 3 |
| 4283 | CXCL9 | C-X-C motif chemokine ligand 9 |
| 4319 | MMP10 | matrix metallopeptidase 10 |
| 4624 | MYH6 | myosin heavy chain 6 |
| 4818 | NKG7 | natural killer cell granule protein 7 |
| 5540 | NPY4R | neuropeptide Y receptor Y4 |
| 6273 | S100A2 | S100 calcium binding protein A2 |
| 6352 | CCL5 | C-C motif chemokine ligand 5 |
| 6846 | XCL2 | X-C motif chemokine ligand 2 |
| 6866 | TAC3 | tachykinin precursor 3 |
| 6947 | TCN1 | transcobalamin 1 |
| 8302 | KLRC4 | killer cell lectin like receptor C4 |
| 8416 | ANXA9 | annexin A9 |
| 9033 | PKD2L1 | polycystin 2 like 1, transient receptor potential cation channel |
| 9047 | SH2D2A | SH2 domain containing 2A |
| 9437 | NCR1 | natural cytotoxicity triggering receptor 1 |
| 9620 | CELSR1 | cadherin EGF LAG seven-pass G-type receptor 1 |
| 10563 | CXCL13 | C-X-C motif chemokine ligand 13 |
| 10578 | GNLY | granulysin |
| 26499 | PLEK2 | pleckstrin 2 |
| 30009 | TBX21 | T-box transcription factor 21 |
| 54967 | CT55 | cancer/testis antigen 55 |
| 56097 | PCDHGC5 | protocadherin gamma subfamily C, 5 |
| 56098 | PCDHGC4 | protocadherin gamma subfamily C, 4 |
| 56100 | PCDHGB6 | protocadherin gamma subfamily B, 6 |
| 56105 | PCDHGA11 | protocadherin gamma subfamily A, 11 |
| 56106 | PCDHGA10 | protocadherin gamma subfamily A, 10 |
| 56120 | PCDHGB8P | protocadherin gamma subfamily B, 8 pseudogene |
| 56121 | PCDHB15 | protocadherin beta 15 |
| 56122 | PCDHB14 | protocadherin beta 14 |
| 56124 | PCDHB12 | protocadherin beta 12 |
| 56127 | PCDHB9 | protocadherin beta 9 |
| 56895 | AGPAT4 | 1-acylglycerol-3-phosphate O-acyltransferase 4 |
| 59067 | IL21 | interleukin 21 |
| 78999 | LRFN4 | leucine rich repeat and fibronectin type III domain containing 4 |
| 79156 | PLEKHF1 | pleckstrin homology and FYVE domain containing 1 |
| 84033 | OBSCN | obscurin, cytoskeletal calmodulin and titin-interacting RhoGEF |
| 85301 | COL27A1 | collagen type XXVII alpha 1 chain |
| 121643 | FOXN4 | forkhead box N4 |
| 126520 | PLK5 | polo like kinase 5 (inactive) |
| 140767 | NRSN1 | neurensin 1 |
| 153769 | SH3RF2 | SH3 domain containing ring finger 2 |
| 162461 | TMEM92 | transmembrane protein 92 |
| 164118 | TTC24 | tetratricopeptide repeat domain 24 |
| 256957 | HEATR9 | HEAT repeat containing 9 |
| 257101 | ZNF683 | zinc finger protein 683 |
| 401427 | OR2A7 | olfactory receptor family 2 subfamily A member 7 |
| 497189 | TIFAB | TIFA inhibitor |
| 730249 | ACOD1 | aconitate decarboxylase 1 |
| TMB per megabase | | |
| TNB per megabase | | |

**Table S5.** The list of common features between the best-performing models using gene expression profile (GEP) and integrated gene expression profiles and clinical data (GEP + clinical) from validation and merged analyses.


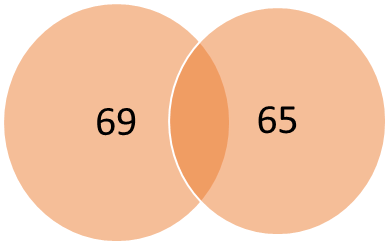


24

**GEP + TMB + TNB (LR-OMC)**

**Validation dataset**

**GEP + TMB + TNB (CART-OMC)**

**Merged datasets**


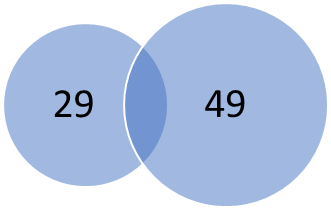


19

**GEP (CART-OMC)**

**Validation analyses**

**GEP (LGBM-OMC)**

**Merged analyses**

| **GEP** | **GEP + clinical** |
| --- | --- |
| CXCL10 | AUNIP |
| CXCL13 | CBX2 |
| CXCL9 | CPLX4 |
| FOXN4 | CXCL13 |
| GNLY | CXCL9 |
| IFNG | CXCR3 |
| IL21 | ESPL1 |
| ITGAE | GABRA3 |
| KCNJ10 | GIPC2 |
| KIR2DL4 | GNLY |
| KLRC2 | GZMM |
| KLRC4 | IFNG |
| LAG3 | IL21 |
| MMP10 | ITGAE |
| NCR1 | KCNJ10 |
| NPY4R | KIR2DL4 |
| NRSN1 | NCR1 |
| S100A2 | NKG7 |
| ZNF683 | NRSN1 |
|  | OIP5 |
|  | PKD2L1 |
|  | TBX21 |
|  | TMB per megabase |
|  | TNB per mega base |

**Table S6**. The Wikipathway cancer and GO pathway enrichment analysis of 29 predictive genes from the best-performing models (CART-OMC) in predicting atezolizumab response in mUC patients (a hypergeometric test corrected with Benjamin-Hochberg).

| **Reference database** | **Enriched pathway** | **#gene in**  **the pathway** | **#gene overlap** | **Gene represented in the pathway** | **Enrichment ratio** | ***P*-Value** | **FDR** |
| --- | --- | --- | --- | --- | --- | --- | --- |
| **Wikipathway cancer** | Type II interferon signaling (IFNG) | 37 | 3 | CXCL10, CXCL9, IFNG | 24.865 | 0.000 | 0.011 |
| **GO: biological process** | cellular defense response | 55 | 6 | CXCL9, GNLY, KIR2DL4, KLRC2, KLRC3, NCR1 | 75.745 | 0.000 | 0.000 |
|  | positive regulation of natural killer cell mediated immunity | 32 | 3 | IL21, KIR2DL4, LAG3 | 65.094 | 0.000 | 0.008 |
|  | regulation of natural killer cell mediated cytotoxicity | 43 | 4 | IL21, KIR2DL4, LAG3, NCR1 | 64.589 | 0.000 | 0.001 |
|  | regulation of natural killer cell mediated immunity | 45 | 4 | IL21, KIR2DL4, LAG3, NCR1 | 61.719 | 0.000 | 0.001 |
|  | natural killer cell mediated immunity | 64 | 5 | IL21, KIR2DL4, KLRC2, LAG3, NCR1 | 54.245 | 0.000 | 0.000 |
|  | natural killer cell mediated cytotoxicity | 60 | 4 | IL21, KIR2DL4, LAG3, NCR1 | 46.289 | 0.000 | 0.002 |
|  | antimicrobial humoral immune response mediated by antimicrobial peptide | 67 | 4 | CXCL10, CXCL13, CXCL9, GNLY | 41.453 | 0.000 | 0.002 |
|  | regulation of cell killing | 88 | 5 | IFNG, IL21, KIR2DL4, LAG3, NCR1 | 39.451 | 0.000 | 0.000 |
|  | regulation of leukocyte mediated cytotoxicity | 72 | 4 | IL21, KIR2DL4, LAG3, NCR1 | 38.574 | 0.000 | 0.003 |
|  | positive regulation of cell killing | 59 | 3 | IFNG, IL21, LAG3 | 35.305 | 0.000 | 0.032 |
|  | leukocyte mediated cytotoxicity | 102 | 4 | IL21, KIR2DL4, LAG3, NCR1 | 27.229 | 0.000 | 0.008 |
|  | cell killing | 157 | 6 | GNLY, IFNG, IL21, KIR2DL4, LAG3, NCR1 | 26.535 | 0.000 | 0.000 |
|  | antimicrobial humoral response | 110 | 4 | CXCL10, CXCL13, CXCL9, GNLY | 25.248 | 0.000 | 0.009 |
|  | regulation of lymphocyte mediated immunity | 142 | 4 | IL21, KIR2DL4, LAG3, NCR1 | 19.559 | 0.000 | 0.019 |
|  | lymphocyte mediated immunity | 238 | 5 | IL21, KIR2DL4, KLRC2, LAG3, NCR1 | 14.587 | 0.000 | 0.010 |
|  | humoral immune response | 242 | 5 | CXCL10, CXCL13, CXCL9, GNLY, IFNG | 14.346 | 0.000 | 0.010 |
|  | regulation of immune effector process | 381 | 6 | IFNG, IL21, KIR2DL4, LAG3, NCR1, ZNF683 | 10.934 | 0.000 | 0.008 |
|  | regulation of immune response | 909 | 8 | CXCL13, GPLD1, IFNG, IL21, KIR2DL4, LAG3, NCR1, ZNF683 | 6.111 | 0.000 | 0.012 |
|  | defense response | 1518 | 13 | CXCL10, CXCL13, CXCL9, GNLY, IFNG, IL21, KIR2DL4, KLRC2, KLRC3, LAG3, NCR1, TTBK1, ZNF683 | 5.946 | 0.000 | 0.000 |
|  | innate immune response | 827 | 7 | IFNG, IL21, KIR2DL4, KLRC2, LAG3, NCR1, ZNF683 | 5.877 | 0.000 | 0.045 |
|  | positive regulation of immune system process | 979 | 8 | CXCL10, CXCL13, GPLD1, IFNG, IL21, KIR2DL4, LAG3, TTBK1 | 5.674 | 0.000 | 0.019 |
|  | immune effector process | 1141 | 9 | CXCL10, CXCL9, IFNG, IL21, KIR2DL4, KLRC2, LAG3, NCR1, ZNF683 | 5.477 | 0.000 | 0.009 |
|  | regulation of immune system process | 1400 | 10 | CXCL10, CXCL13, GPLD1, IFNG, IL21, KIR2DL4, LAG3, NCR1, TTBK1, ZNF683 | 4.960 | 0.000 | 0.008 |
|  | immune response | 1919 | 12 | CXCL10, CXCL13, CXCL9, GNLY, GPLD1, IFNG, IL21, KIR2DL4, KLRC2, LAG3, NCR1, ZNF683 | 4.342 | 0.000 | 0.003 |
| **GO: molecular function** | CXCR3 chemokine receptor binding | 5 | 3 | CXCL10, CXCL13, CXCL9 | 476.230 | 0.000 | 0.000 |
|  | CXCR chemokine receptor binding | 11 | 3 | CXCL10, CXCL13, CXCL9 | 216.470 | 0.000 | 0.001 |
|  | chemokine activity | 47 | 3 | CXCL10, CXCL13, CXCL9 | 50.663 | 0.000 | 0.025 |
|  | chemokine receptor binding | 61 | 3 | CXCL10, CXCL13, CXCL9 | 39.035 | 0.000 | 0.046 |
|  | cytokine activity | 217 | 5 | CXCL10, CXCL13, CXCL9, IFNG, IL21 | 18.288 | 0.000 | 0.010 |
|  | cytokine receptor binding | 274 | 5 | CXCL10, CXCL13, CXCL9, IFNG, IL21 | 14.484 | 0.000 | 0.022 |
| **GO: cellular component** | external side of plasma membrane | 289 | 5 | CXCL10, CXCL9, ITGAE, LAG3, RTBDN | 16.910 | 0.000 | 0.016 |
